# Superior Resolution Profiling of the *Coleofasciculus* Microbiome by Amplicon Sequencing of the Complete 16S rRNA Gene and ITS Region

**DOI:** 10.1111/1758-2229.70066

**Published:** 2025-01-31

**Authors:** Pia Marter, Heike M. Freese, Victoria Ringel, Henner Brinkmann, Silke Pradella, Manfred Rohde, Michal Jarek, Cathrin Spröer, Irene Wagner‐Döbler, Jörg Overmann, Boyke Bunk, Jörn Petersen

**Affiliations:** ^1^ Leibniz Institute DSMZ – German Collection of Microorganisms and Cell Cultures Braunschweig Germany; ^2^ Helmholtz Centre for Infection Research Braunschweig Germany; ^3^ Institute of Microbiology, Technical University of Braunschweig Braunschweig Germany

**Keywords:** 16S‐ITS, cyanosphere, isolates, long‐read, marine, metagenome binning, PacBio amplicon sequencing

## Abstract

The filamentous cyanobacterium *Coleofasciculus chthonoplastes* is the key primary producer of marine microbial mats. We elucidated the microbiomes of 32 non‐axenic *Coleofasciculus* isolates using PacBio‐based amplicon sequencing of the complete 16S rRNA gene and the internally transcribed spacer (16S‐ITS). The length of authentic amplicon sequence variants (ASVs) ranged from 1827 to 3044 nucleotides (median: 2267 nt). The results, which were complemented by metagenome analyses and cultivation approaches, revealed the presence of more than 70 associated heterotrophs in the culture of *Coleofasciculus* sp. WW12. The great bacterial diversity in the cyanosphere is dominated by *Pseudomonadota* (59%) and *Bacteroidota* (23%). Allelic ribosomal operon variants were detected in 18 *Coleofasciculus* strains and our analyses proposed the presence of at least four different species. A comparative analysis of cyanobacterial microbiomes documented complementary advantages of amplicon sequencing versus metagenomics with an individual strength of the 16S‐ITS approach in terms of (i) ribosomal target sequence quality, (ii) contaminant detection and (iii) identification of rare bacteria. The characterisation of the *Coleofasciculus* microbiome showed that long‐read amplicon sequencing of the 16S‐ITS region is the method of choice for rapid profiling of non‐axenic cyanobacteria. Its superior resolution allows a reliable differentiation of even very closely related strains.

## Graphical abstract

High‐throughput amplicon sequencing of the 16S rRNA gene and the adjacent internally transcribed spacer region (16S‐ITS) on the PacBio platform allows a rapid characterisation of non‐axenic cyanobacteria and their microbiomes on strain level. The detection of ribosomal operon variants reflects the unprecedented resolution of the novel long‐read sequencing approach.

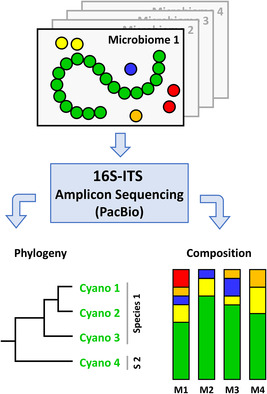

**Abbreviations**
ANI: average nucleotide identity
ASV: amplicon sequence variant
BC: barcode
CCS: circular consensus sequencing
dDDH: digital DNA–DNA hybridization
DSMZ: Deutsche Sammlung von Mikroorganismen und Zellkulturen
GTDB: genome taxonomy database
ITS: internally transcribed spacer
MAG: metagenome‐assembled genome
NCBI: National Center of Biotechnological Information
PacBio: Pacific Biosciences
SEM: scanning electron microscopy
SRA: sequence read archive
TYGS: type (strain) genome server

## 1 Introduction

The morphological diversity of cyanobacteria, ranging from uniform cocci to filamentous strains with highly specialised cell types, is unprecedented in prokaryotic evolution and was the basis for their classification for more than a century. Molecular analyses of marker genes and complete genomes revealed the limitations of this botanical approach, and today the 16S rRNA gene is the gold standard for the initial characterisation of new strains. However, unlike typical bacterial isolates, which are pure cultures, the initially non‐axenic state of new cyanobacterial strains impedes their rapid classification. Routine PCR amplification with universal primers results in a mixture of amplicons that cannot be sequenced directly with the Sanger technique. This problem was solved by the use of specific primers (Wilmotte, Van der Auwera, and De Wachter 1993; Nübel, Garcia‐Pichel, and Muyzer 1997), and the current characterisation of cyanobacteria and algae is still based on PCR methods developed three decades ago (Boyer, Flechtner, and Johansen 2001; Mikhailyuk et al. 2019; Brenes‐Guillén et al. 2021; Jung et al. 2021; Keshari et al. 2022). However, the classical approach is often fraught with PCR problems, including partial 16S rDNA amplificates, Sanger sequences of comparably poor quality, and the requirement of unicyanobacterial isolates. Next generation amplicon sequencing on the Illumina platform, which is widely used for environmental studies (Strunecký et al. 2019; Glaser et al. 2022; Keshari et al. 2022), allows only a comparably coarse taxonomic resolution on the genus level due to the short sequence length of the targeted regions of the 16S rRNA gene (V3‐V4: 300 bp; V4‐V5: 400 bp; V5‐V6: 200 bp). An alternative to overcome these technical limitations is long‐read amplicon sequencing on the PacBio platform (Fichot and Norman 2013), which allows resolution down to the level of a single deviating nucleotide (Callahan et al. 2019). Therefore, here we used high‐throughput PacBio amplicon sequencing of the nearly complete 16S‐ITS region and established an analysis pipeline to generate superior resolution sequence data for both the cyanobacterial isolate and the accompanying heterotrophic microbiome. The ITS region was included because it has been shown to be an important region for the taxonomic classification of cyanobacteria (Martins, Machado‐de‐Lima, and Branco 2019; Zhang et al. 2022; Carmona Jiménez et al. 2023; Skoupý et al. 2024). PacBio amplicon sequencing of the 16S rRNA gene has recently been used to analyse the microdiversity of bacteria in pelagic freshwater systems, soil agroecosystems, anaerobic digesters, and the faecal microbiome (Lam et al. 2020; Graf et al. 2021; Okazaki et al. 2021; He et al. 2022), but the current study is, to our knowledge, the first to characterise the microbiome of non‐axenic cyanobacteria.

**FIGURE 1:**
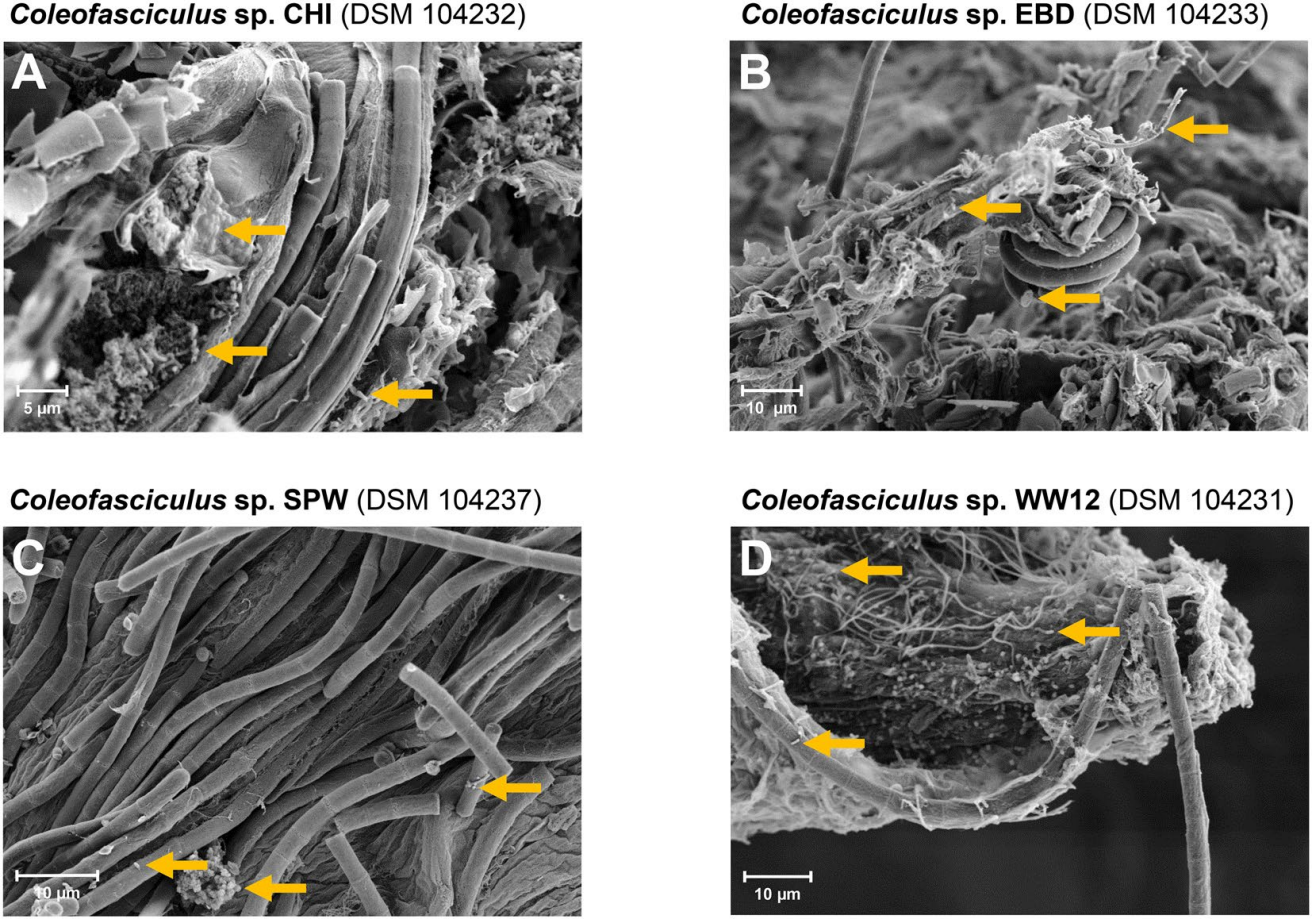
Scanning electron microscopy of four non‐axenic cyanobacteria of the genus *Coleofasciculus* (CHI, EBD, SPW, WW12). Associated heterotrophic bacteria, which are very small compared to the cyanobacterium, are highlighted with orange arrows.

The genus *Coleofasciculus* is the phototrophic key taxon of marine microbial mats (Siegesmund et al. 2008). Microbial mats are vertically laminated macroscopic accumulations of functionally highly diverse microorganisms with a coherent structure, which can be traced back to physicochemical gradients generated by their metabolic activity (van Gemerden 1993; Des Marais 1995; Bolhuis, Cretoiu, and Stal 2014). Numerous studies based on samples collected worldwide from salt marshes and tidal flats have shown that these stratified microbial communities are dominated by the cyanobacterium *Coleofasciculus* (Stolz 1985; Jørgensen, Cohen, and Revsbech 1986; Visscher and Van Gemerden 1991; Karsten 1996; Nübel et al. 2000; Lodders, Stackebrandt, and Nübel 2005; Tkavc et al. 2011; Lee et al. 2018; Vogt et al. 2019). Their interwoven and attached filaments (Figure 1) are the basis for the solid composition of microbial mats that are important for the stabilisation of the sediment surface, terraforming and coast protection (Bolhuis, Cretoiu, and Stal 2014). Layered marine microbial sediments are often considered as modern forms of fossilised stromatolites (Margulis et al. 1980; van Gemerden 1993). Contemporary equivalents of stromatolites with filamentous cyanobacteria closely related to *Coleofasciculus* sp. PCC 7420 have been found in shallow saline habitats in Western Australia (Papineau et al. 2005), but also in a high‐altitude volcanic lake in the Argentine Andes (Toneatti et al. 2017). Regardless of the general occurrence of *Coleofasciculus*, two studies showed a remarkable dynamic of the composition of the microbial mat with an annual succession of the cyanobacterial primary producer ranging from 0.1% in spring to more than 10% in fall (Cardoso et al. 2019; Jazmín et al. 2023). The characterisation of the heterotrophic community associated with *Coleofasciculus* is therefore a promising first step for characterising the ecology of these microbial mats.

*Coleofasciculus* strains of the current study were isolated from various marine habitats in Europe, North and South America, Asia and Australia between 1993 and 2003. The worldwide largest collection of 32 non‐axenic isolates (Table 1) is deposited in the German Collection of Microorganisms and Cell Cultures (DSMZ). The unbranched, filamentous non‐heterocystous cyanobacteria, which are morphologically hardly distinguishable from distantly related genera such as *Microcoleus*, *Leptolyngbya* or *Trichocoleus*, have been identified since the 1990s on the basis of their 16S rRNA gene and adjacent internally transcribed spacer (ITS) region (Boyer et al. 2002; Siegesmund et al. 2008). *Coleofasciculus chthonoplastes* WW7^T^ is the type strain of the only described species of the genus, but phylogenetic 16S‐ITS analyses suggested the presence of another morphologically indistinguishable cryptic *Coleofasciculus* species based on the separation of strains into two closely related subtrees (Siegesmund et al. 2008). The first genome‐sequenced strain PCC 7420 from the Pasteur Culture Collection is axenic, but the majority of isolates comprise a complex but as yet uncharacterized community of associated heterotrophic bacteria.

**TABLE 1:** List of 32 *Coleofasciculus* and two *Salileptolyngbya* strains of the DSMZ culture collection investigated in the current study.

	Strain	Culture ID	Sub.	Unicyano.	Axenic	Sampling site	Location	Country	Year	BioProject	Ref.
00	x	PCC 7420	I	Yes	Yes	Salt marsh	Sippewisset, Woods Hole	USA (MA)	1974	PRJNA19325	Garcia‐Pichel, Prufert‐Bebout, and Muyzer 1996
01	NCR	DSM 104235	I	Yes	No	Intertidal	Rachel Carson Estuarine Reserve	USA (NC)	1993	PRJNA993010	Garcia‐Pichel, Prufert‐Bebout, and Muyzer 1996
02	**SPW**	DSM 104237	I	No	No	Salt marsh	Sippewisset, Woods Hole	USA (MA)	1993	PRJNA993097	Siegesmund et al. 2008
03	**EBD**	DSM 104233	I	Yes	No	Hypersaline pool	Ebro Delta	Spain	1993	PRJNA993099	Garcia‐Pichel, Prufert‐Bebout, and Muyzer 1996
04	WW3	DSM 104222	I	No	No	Wind flat	Zingst	Germany	2002/2003	PRJNA993103	Siegesmund et al. 2008
05	BRE	DSM 104253	I	Yes	No	Salt marsh	Sables D'Or, Brittany	France	2001	PRJNA993106	Siegesmund et al. 2008
06	WW6	DSM 104225	I	Yes	No	Wind flat	Zingst	Germany	2002/2003	PRJNA993108	Siegesmund et al. 2008
07	WW5	DSM 104224	I	Yes	No	Wind flat	Zingst	Germany	2002/2003	PRJNA993113	Siegesmund et al. 2008
08	SA18	DSM 104244	I	Yes	No	x	Dawhat al‐Musallamiya, Jubail	Saudi Arabia	1995	PRJNA993115	Siegesmund et al. 2008
09	**CHI**	DSM 104232	I	Yes	No	Intertidal	Dichato Marine Station	Chile	1994	PRJNA993444	Karsten 1996
10	GNL1	DSM 104238	I	Yes	No	Intertidal lagoon	Guerrero Negro	Mexico	1993	PRJNA993446	Garcia‐Pichel, Prufert‐Bebout, and Muyzer 1996
11	SAH	DSM 104254	I	Yes	No	Salzhaff	x	Germany	2002	PRJNA993447	Siegesmund et al. 2008
12	VOE	DSM 104245	I	Yes	No	Intertidal	Voersa	Denmark	2002	PRJNA993448	x
13	WW7^T^	DSM 106952^a^	I	Yes	No	Wind flat	Zingst	Germany	2002/2003	PRJNA993450	Siegesmund et al. 2008
14	MAF	DSM 104249	I	Yes	No	Brackish	Mariager Fjord	Denmark	2002	PRJNA993452	Siegesmund et al. 2008
15	WW11	DSM 104230	I	Yes	No	Wind flat	Zingst	Germany	2002/2003	PRJNA993453	Siegesmund et al. 2008
16	DO3	DSM 104240	I	Yes	No	Wind flat	Darßer Ort	Germany	2005	PRJNA993455	x
17	STO	DSM 104242	I	Yes	No	Sediment	St. Peter Ording	Germany	1995	PRJNA993456	Siegesmund et al. 2008
18	TOW	DSM 104236	I	Yes	No	Mangroves	Towra Point, Sydney	Australia	1993	PRJNA993458	Karsten 1996
19	NDN	DSM 104251	I	Yes	No	x	Norderney	Germany	1993	PRJNA993459	Karsten and Garcia‐Pichel 1996
20	GNP5	DSM 104239	I	Yes	No	Intertidal saline	Guerrero Negro	Mexico	1993	PRJNA993461	Siegesmund et al. 2008
21	SOL	DSM 104241	I	Yes	No	Hypersaline pond	Solar Lake, Sinai peninsula	Egypt	1993	PRJNA993462	Garcia‐Pichel, Prufert‐Bebout, and Muyzer 1996
22	**WW12**	DSM 104231	II	Yes	No	Wind flat	Großer Werder, Zingst	Germany	2002/2003	PRJNA993463	Siegesmund et al. 2008
23	WW1	DSM 104220	II	Yes	No	Wind flat	Zingst	Germany	2002/2003	PRJNA993465	Siegesmund et al. 2008
24	WW2	DSM 104221	II	Yes	No	Wind flat	Zingst	Germany	2002/2003	PRJNA993467	Siegesmund et al. 2008
25	WW9	DSM 104228	II	Yes	No	Wind flat	Zingst	Germany	2002/2003	PRJNA993468	Siegesmund et al. 2008
26	WW10	DSM 104229	II	Yes	No	Wind flat	Zingst	Germany	2002/2003	PRJNA993469	Siegesmund et al. 2008
27	CCY0002	DSM 101195	II	Yes	Yes	x	Schiermonnikoog	Netherlands	x	PRJNA993470	Bolhuis et al. 2010
28	EDA	DSM 104234	II	Yes	No	Brackish	Eldena	Germany	2002	PRJNA993471	Siegesmund et al. 2008
29	ASK1	DSM 104255	II	Yes	No	Brackish	Askø	Sweden	2002	PRJNA993479	Siegesmund et al. 2008
30	ASK5	DSM 104256	II	Yes	No	Brackish	Askø	Sweden	2002	PRJNA993480	Siegesmund et al. 2008
31	LZW	DSM 104248	II	Yes	No	Brackish	Lietzow, Jasmunder Bodden	Germany	2002	PRJNA993481	Siegesmund et al. 2008
32	WIS	DSM 101416	II	Yes	No	Brackish sediment	Poel Island	Germany	1994	PRJNA993482	Karsten 1996
33	WW4	DSM 104223	C3	Yes	No	Wind flat	Zingst	Germany	2002/2003	PRJNA993484	Siegesmund et al. 2008
34	WW8	DSM 104227	C3	Yes	No	Wind flat	Zingst	Germany	2002/2003	PRJNA993486	Siegesmund et al. 2008

*Note:* Genome‐sequenced strains are highlighted in bold with a grey background. *Salileptolyngbya* strains are shown in grey. Sub., phylogenetic subtree of the genus *Coleofasciculus* (Figure 4).

Abbreviations: unicyano, unicyanobacterial; Ref, reference.

^a^ Type strain of *Coleofasciculus chthonoplastes* (DSM 106952 = SAG 2209).

In the current study we investigated 32 non‐axenic *Coleofasciculus* strains via 16S‐ITS amplicon sequencing, which allowed superior resolution profiling of very closely related strains of this cosmopolitan cyanobacterium. Furthermore, co‐amplification of the associated heterotrophic bacteria provided insights into the composition of their cyanosphere at a high taxonomic resolution. Short‐read metagenome sequencing of four strains, selected due to the high diversity of their cyanosphere, allowed us to compare the advantages and limitations of amplicon versus metagenome sequencing. Finally, we were able to isolate heterotrophic inhabitants of the cyanosphere that were dominant and present in many *Coleofasciculus* cultures as shown by our 16S‐ITS sequencing pipeline.

## 2 Materials and Methods

### 2.1 Origin and Cultivation of Cyanobacteria

The 32 investigated filamentous *Coleofasciculus* strains deposited at the DSMZ (German Collection of Microorganisms and Cell Cultures, Braunschweig, Germany) originated from marine and saline habitats (Table 1). The two other strains examined (WW4, WW8) were previously misclassified as *Coleofasciculus* sp. (Siegesmund et al. 2008), but belong to the genus *Salileptolyngbya* (see current study). Strains were cultivated in 20 mL cyanobacteria medium MCL (DSMZ medium 1680), BA+ (DSMZ medium 1677), SWES (DSMZ medium 1831) or ASN3+ (DSMZ medium 1673) in 25 cm^2^ tissue culture flasks (Techno Plastic Products AG [TPP], Trasadingen, Switzerland). All strains were continuously cultivated at 17°C under low light conditions (3–4 mol s^−1^ m^−2^) with a combination of three fluorescent bulbs (Osram L30W/830 Lumilux warm white, Osram L30W/840 Lumilux cool white, Osram L30W/77 Fluora) at a day/night cycle of 16/8 h. 5 mL of the culture was transferred to fresh medium every one to 3 months.

### 2.2 Electron Microscopy

For scanning electron microscopy (SEM) with the Zeiss Merlin field emission scanning electron microscope (Carl Zeiss, Oberkochen, Germany), cyanobacteria were fixed in the cultivation medium with glutaraldehyde (final concentration 2%). The final fixation was achieved after 30 min by adding formaldehyde (Riedel‐de Haën, Seelze, Germany) to a final concentration of 5%. SEM was performed as previously described (Will et al. 2019).

### 2.3 High‐Throughput PacBio 16S‐ITS Amplicon Sequencing

DNA extraction of cyanobacterial DNA with the DNeasy Blood and Tissue Kit (Qiagen, Hilden, Germany) was conducted as recently described (Marter et al. 2021). DNA from cyanobacterial cultures was isolated in 2019 and again in 2023 (34 ng—18.6 μg) and reproducibility of the sequencing approach was tested by sequencing of up to four independent amplicon libraries per culture. PCR amplification of the 16S rRNA gene and the adjacent internally transcribed spacer (ITS) region was performed with the barcoded 16S rRNA forward primer (16S_27f: 5′‐AGAGTTTGATCMTGGCTCAG‐3′; [Suzuki and Giovannoni 1996]) and the 23S rRNA reverse primer (23S_130r: 5′‐GGGTTBCCCCATTCRG‐3′; [Hunt et al. 2006]). The usage of individual PacBio barcodes (see Table S1A) allows parallel sequencing of up to 96 different amplicons per sequencing run. We tested several DNA polymerases and finally used the enzyme with the highest fidelity, that is, the Platinum SuperFi DNA polymerase (Invitrogen), to minimise the number of PCR errors. PCR reactions were performed in a total volume of 25.0 μL with 1.0 μL of DNA template (1.0 ng), 1.25 μL of each barcoded primer (10 μM), 9.0 μL dH_2_O, and 12.5 μL of the Invitrogen Platinum SuperFi PCR Master Mix. PCR amplification in the Eppendorf Mastercycler Gradient 5331 was conducted with 30 cycles and an extension time of 1:30 min, which allowed long‐range amplification of 16S‐ITS sequences of more than 3 kb ([i] 1× initial denaturation: 30 s, 98°C; [ii] 30× denaturation: 10 s, 98°C, annealing: 10 s, 52°C, extension: 1:30 min, 72°C; [iii] 1× final extension: 5 min, 72°C). Amplification of 16S‐ITS sequences was visually confirmed by agarose gel electrophoresis of 2 μL aliquots. Amplicons were then purified with the QIAquick PCR Purification Kit (Qiagen) and DNA concentrations between 5.7 and 65.4 ng/μL were measured with the NanoDrop spectrophotometer. More than 50 amplicons with individual barcodes were equimolarly pooled and 5000 ng of DNA, which is 10 times the minimum amount, were used for library preparation.

SMRTbell template library was prepared according to the instructions from Pacific Biosciences (PacBio), Menlo Park, CA, USA, following the Procedure & Checklist—Preparing multiplexed amplicon libraries using SMRTbell prep kit 3.0 (PN 102‐359‐000 REV 02 SEP2022). Briefly, amplicons were end‐repaired and ligated to barcoded adapters applying components from the SMRTbell Prep Kit 3.0 from Pacific Biosciences. Reactions were carried out according to the manufacturer's instructions. Conditions for annealing of sequencing primers and binding of polymerase to purified SMRTbell template were assessed with the Calculator in SMRTlink (Pacific Biosciences).

Libraries were sequenced on the PacBio Sequel IIe taking one 10 h movie per SMRT cell. Highly accurate single‐molecule consensus reads (HiFi reads) were obtained using the circular consensus sequencing (CCS) mode on the PacBio long‐read system. Amplicon data was demultiplexed using the demultiplex barcodes application within SMRTlink 11. Hereby, a minimum CCS predicted accuracy of 20 as well as a minimum barcode score of 80 was applied using the symmetric barcoding option.

### 2.4 Analysis Pipeline of 16S‐ITS Amplicon Sequences

The circular consensus sequence reads were analysed using the slightly modified DADA2 pipeline in R 4.2.1. with the default parameters if not otherwise specified (Callahan et al. 2016). In short, sequences that did not contain both primers were discarded and the primer sequences were removed from the remaining sequences. Sequences with a length of less than 1400 bp or those that did not meet the default filter parameters were also discarded, but none was truncated. All sequences were then dereplicated, denoised and chimaeras were removed (using minParentAbundance = 6). For taxonomic classification, the 16S rDNA was extracted from the complete ASVs by mapping and cutting before the 16S_1492r primer sequence on the plus strand (5′‐AGTCGTAACAAGGTARCC‐3′; [Suzuki and Giovannoni 1996]). The classification was based on the SILVA reference database (SILVA 138.1; [Quast et al. 2013]). ASVs were accepted as true biological variants if they occurred in at least two independent amplicon sequencing experiments of the same strain. Cyanobacterial ASVs, which could only be detected in one experiment were investigated individually to exclude amplification or sequencing artefacts. All authentic and additional manually confirmed cyanobacterial ASVs were deposited at the NCBI database (GenBank accession numbers OR000000; see Table S1).

### 2.5 Metagenome Sequencing, Binning, and Classification

DNA extracted in 2019 from *Coleofasciculus* sp. CHI (2214 ng), EBD (88 ng), SPW (2401 ng), and WW12 (706 ng) were used for metagenome sequencing as previously described (Marter et al. 2021). Illumina libraries were prepared using the NEBNext Ultra II FS DNA Library Prep Kit (New England Biolabs, Frankfurt, Germany). Paired‐end sequencing of the libraries (PE 150) with two times 100 million reads per sample was performed on the Illumina NovaSeq 6000 system using the v3 chemistry (600 cycles). Quality control and adapter clipping of the sequences was done using the fastq‐mcf tool of ea‐utils v1.04.803. Filtered and trimmed raw sequence data were uploaded to NCBI's Sequence Read Archive (Accession numbers: SRR26209547, SRR26209553, SRR26209612, and SRR26147846).

Metagenome assembly and binning was conducted using the pipeline developed by Marter et al. (2021) as follows. Reads were assembled with MEGAHIT v1.2.7 (Li et al. 2015), assemblies were binned in parallel with three different methods (MaxBin 2.0 v2.2.6 (Wu, Simmons, and Singer 2016), MetaBAT v2.12.1 (Kang et al. 2015), Concoct v1.1.0 (Alneberg et al. 2014)), and the results were analysed with DAS Tool v1.1.2 (Sieber et al. 2018). Genome coverage was determined by mapping of the raw reads on the final set of metagenome‐assembled genomes (MAGs). The quality of MAGs in terms of completeness and contamination was finally assessed with CheckM v1.0.13 (Parks et al. 2015). The annotated MAGs from the cyanosphere of *Coleofasciculus* are deposited in NCBI GenBank under the accession numbers: SAMN36814870‐901 (Strain CHI, BioProject: PRJNA993444), SAMN36814729‐62 (Strain EBD, BioProject: PRJNA993099), SAMN36798168‐213 (Strain SPW, BioProject: PRJNA993097) and SAMN36815369‐413 (Strain WW12, BioProject: PRJNA993463).

Initial taxonomic classification of MAGs from the current study was conducted with the type (strain) genome server TYGS (Meier‐Kolthoff and Göker 2019) and the Genome Taxonomy Database Toolkit GTDB‐Tk v2.1.0 on the GTDB reference data version r207 (Parks et al. 2022). Their final taxonomic assignment was based on the List of Prokaryotic names with Standing in Nomenclature LPSN (Parte 2018; Meier‐Kolthoff et al. 2022). Word clouds were calculated based on the genome coverage of MAGs and their final classification in R with the wordcloud package.

Genome comparisons were performed with the Genome‐to‐Genome Distance Calculator (GGDC 3.0; (Meier‐Kolthoff et al. 2022)) and the average nucleotide (ANI) calculator using OrthoANIu (Yoon et al. 2017).

### 2.6 Composition of Non‐Axenic Cyanobacteria Cultures

Quantitative taxonomical assessment of 16S‐ITS amplicon sequences was performed at phylum level (*Pseudomonadota* [synonym: *Proteobacteria*]: class level) based on the number of sequence counts from authentic ASVs. The corresponding metagenomic assessment was based on the genome coverages of high‐quality MAGs with a completeness > 80% and a contamination rate < 10%.

Descriptive statistics of associated bacteria from all 32 *Coleofasciculus* cultures examined was conducted based on ASVs detected in at least two independent sequencing experiments. Three cultures that did not contain authentic ASVs from associated bacteria were therefore excluded from the analysis (NCR, ASK1, CCY0002).

### 2.7 Phylogenetic Analyses

16S rRNA gene sequences were aligned with Clustal Omega (Sievers et al. 2011) and all positions with less than 78% site coverage were eliminated. The evolutionary history was inferred by the Maximum Likelihood (ML) method based on the Kimura 2‐parameter model (Kimura 1980). Phylogenetic analyses, with 100 bootstrap replicates, were conducted in MEGA7 v7.0.25 (Kumar, Stecher, and Tamura 2016). Genome‐based trees were reconstructed from concatenated amino acid alignments of 92 housekeeping genes with UBCG (Na et al. 2018). Alignments were manually refined with the MUST package (Philippe 1993) and the application of G‐blocks (Talavera and Castresana 2007). Phylogenomic ML trees with 100 bootstrap replicates were finally calculated with RAxML v8.2.10 (Stamatakis 2014).

### 2.8 Isolation of Heterotrophic Bacteria

100 μL culture supernatant of *Coleofasciculus* sp. WW12 was diluted in 900 μL 0.5× MCL medium (DSMZ medium 1680) in seven steps. 200 μL of dilutions 10^−5^, 10^−6^ and 10^−7^ were plated on agar plates with marine broth (DSMZ medium 514f) and 0.2‐fold marine broth medium (8.0 g MB [Carl Roth: CP73.1], 29.8 g Sea Salts [Sigma: S9883]/litre) and incubated at 28°C. A total number of more than 300 colonies with a focus on different colours and morphologies were further purified. Bacterial isolates were cultivated in 4 mL MB medium at 28°C. DNA extraction was performed using the MasterPure Gram Positive DNA purification kit (Epicentre: MGP04100). PCR amplification of the 16S rRNA gene and the adjacent internally transcribed spacer (ITS) region was performed with the 16S rRNA forward primer (16S_10‐30f [P723]: 5′‐GAGTTTGATCCTGGCTCA‐3′; (Farrelly, Rainey, and Stackebrandt 1995)) and the 23S rRNA reverse primer (23S_241‐256r [P677]: 5′‐KTTCGCTCGCCRCTAC‐3′; (Dewhirst et al. 2005)) or the 16S rRNA reverse primer (16S_1406r [P239]: 5′‐ACGGGCGGTGTGTRCAA‐3′; (Nagashima et al. 2003)). Sanger sequencing of PCR amplificates either with the 16S forward or the 16S reverse primer was performed by LGC Genomics (Berlin, Germany).

## 3 Results and Discussion

### 3.1 Strains and Sequencing Characteristics

Our 16S‐ITS sequencing approach was performed for 32 filamentous cyanobacteria of the genus *Coleofasciculus* deposited in the DSMZ culture collection (Table 1). Two other strains (WW4, WW8) were also previously classified as *Coleofasciculus* (Siegesmund et al. 2008), but the current study has clearly shown that the corresponding cultures deposited at the DSMZ are unicyanobacterial isolates of the genus *Salileptolyngbya* (see below). The strains had been sampled worldwide, with a focus on the Baltic Sea, and originated from the intertidal zone, salt marshes and hypersaline ponds (Garcia‐Pichel, Prufert‐Bebout, and Muyzer 1996; Karsten 1996; Karsten and Garcia‐Pichel 1996; Siegesmund et al. 2008; Bolhuis et al. 2010). Electron micrographs of the four genome‐sequenced *Coleofasciculus* strains (CHI, EBD, SPW, WW12) illustrate their tight association in bundles with sometimes massive sheaths of extracellular matrix and the presence of comparably small associated heterotrophic bacteria (Figure 1). These morphologic characteristics reflect the challenges in isolating unicyanobacterial cultures, explain why most of the strains are non‐axenic and suggest the presence of a unique but completely unknown microbiome. Our high‐throughput sequencing results provided clear evidence that none of the 34 investigated cultures contained cyanobacteria from other genera (Table S1B). A total number of 1,004,435 16S‐ITS PacBio long‐read sequences with a total size of 2.3 Gbp passed our filtering pipeline. 16S‐ITS amplicon sequencing of 34 strains resulted in 943 different ASVs, including a set of 354 authentic ASVs validated by at least two independent sequencing experiments (Table S1B). Their length ranged from 1827 to 3044 nucleotides (median: 2267 nt).

Metagenome sequencing and binning of four strains (CHI, EBD, SPW, WW12) resulted in up to 40 MAGs with completeness greater than 80% and contamination rates less than 10%, with each metagenome containing a single cyanobacterial MAG of the genus *Coleofasciculus* (Table S2). Based on the genome coverage, the cyanobacterium was one of the three most abundant MAGs in the four investigated metagenomes. The current study showed that successful binning depends on at least six‐fold genome coverage.

### 3.2 Reliability of 16S‐ITS Amplicon Sequencing of Non‐Axenic Cyanobacteria

#### 3.2.1 Effect of Primer Barcodes and DNA Sampling on 16S‐ITS Amplicon Sequencing

**FIGURE 2:**
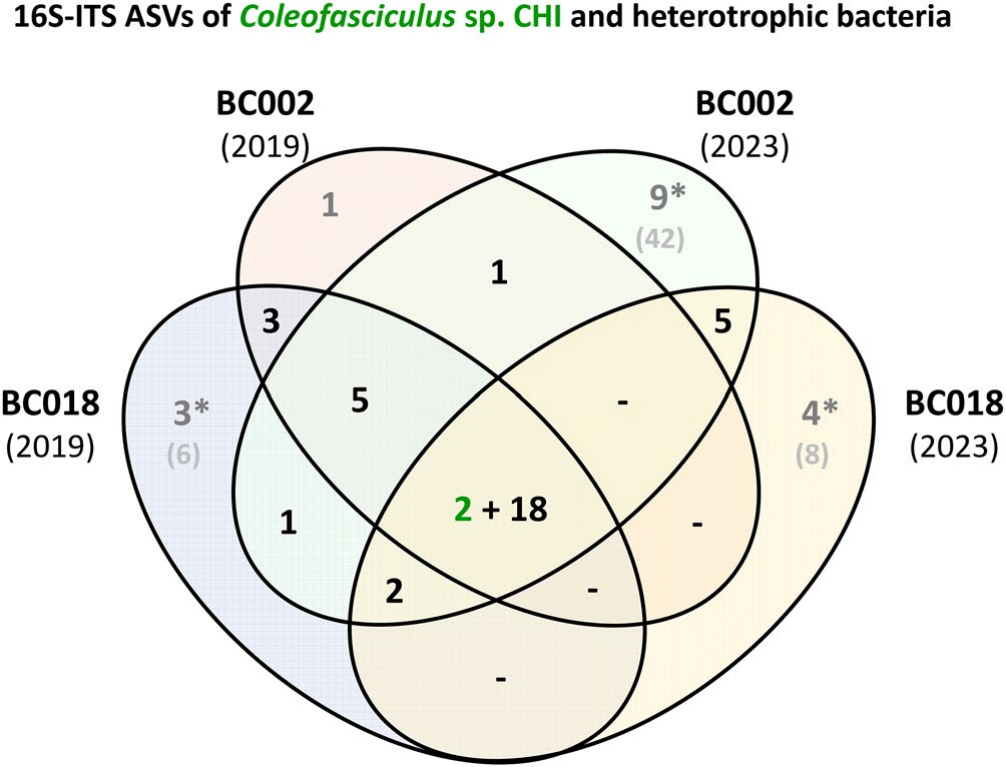
Comparison of the 16S‐ITS PacBio amplicon sequencing approach with four independent replicates. The Venn diagram shows the distribution of Amplicon Sequence Variants (ASVs) established from the non‐axenic cyanobacterium *Coleofasciculus* sp. CHI (= DSM 104232). DNA of the continuously cultivated strain was isolated in 2019 and 2023, PCR amplification was performed with two different primer barcodes (BC002, BC018). Authentic ASVs from *Coleofasciculus* and associated heterotrophic bacteria that were found in at least two replicates are shown in green and black, respectively. ASVs from a single sequencing experiment (= singletons) are shown in grey. *, authentic singletons; 16S‐ITS, 16S rRNA gene and internally transcribed spacer region. Further information regarding the taxonomic classification, 16S‐ITS sequence and abundance of all 94 ASVs is provided in Table S7A.

**FIGURE 3:**
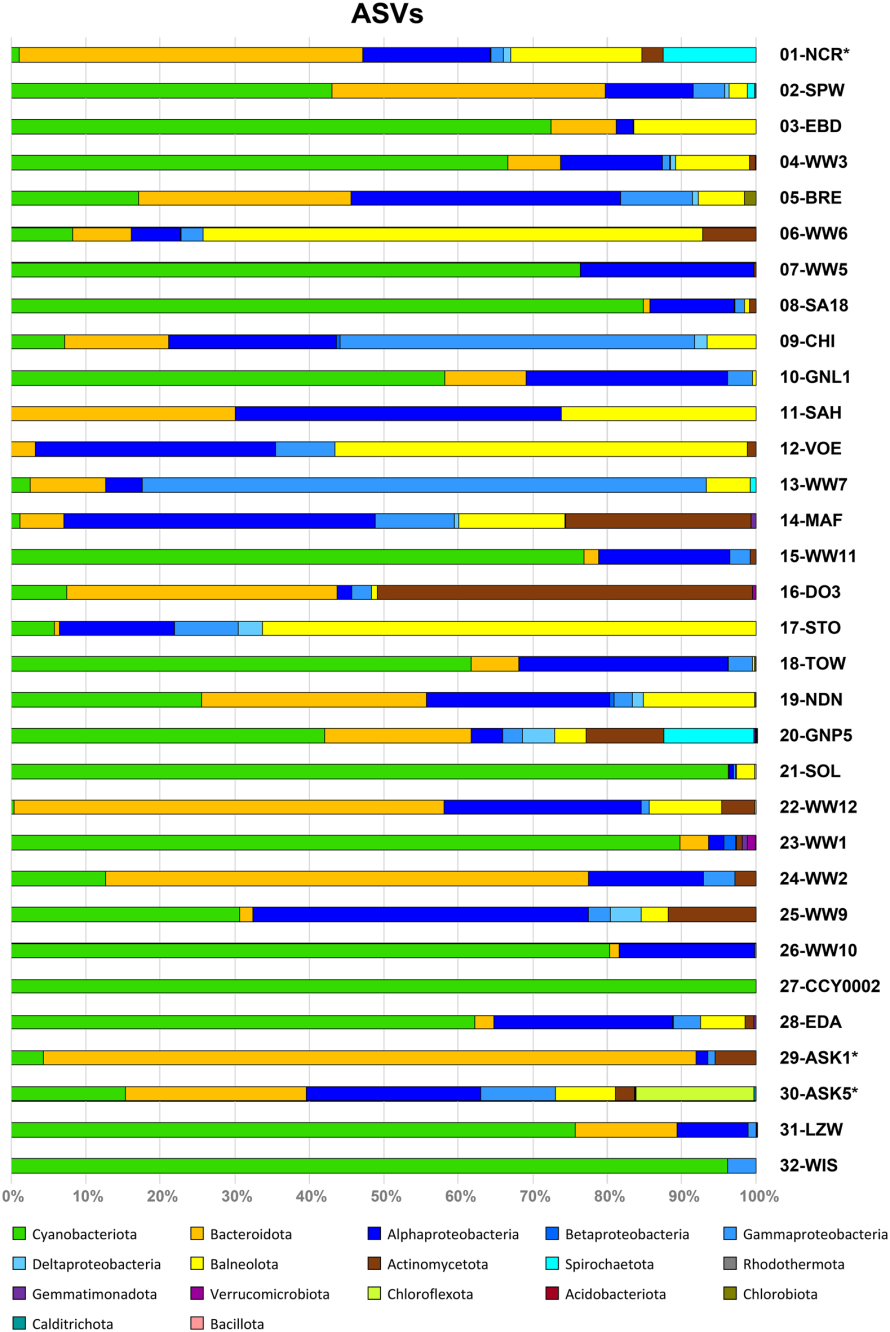
Bacterial composition of 32 non‐axenic *Coleofasciculus* strains based on 16S‐ITS amplicon sequencing. The bar graph shows the averaged counts of authentic ASVs from all sequencing runs (Table S1). * Bar with all ASVs derived from one or two sequencing experiments (NCR: BC048‐2019; ASK1: BC127‐2023; ASK5: BC012‐2019, BC012‐2023).

We compared four independent sequencing experiments of the non‐axenic strain *Coleofasciculus* sp. CHI to investigate the reproducibility and limitations of our newly established pipeline for long‐read amplicon sequencing. We investigated (i) the effects of two different primer barcodes (BC002, BC018) on PCR amplification of the same DNA sample and (ii) the use of two different DNA samples from the same culture of which one was isolated in 2019 and the other one after 4 years of continuous cultivation in 2023. The four runs resulted in a total amount of more than 40,000 16S‐ITS sequences with amplicon sizes ranging from 1827 to 2804 bp (Table S1). These sequences are represented by 94 individual ASVs that differ in at least one nucleotide position. 37 ASVs were obtained at least twice and 20 ASVs were amplified in all four experiments (Figure 2). A comparison of the 37 authentic ASVs showed no systematic bias from barcoding (Text S1). In contrast, the ASV comparison of DNA samples from the same microbial consortium isolated in 2019 and 2023 displayed considerable differences (Figure S1), and the analysis of all 32 *Coleofasciculus* cultures revealed individual fingerprints (Figure 3).

#### 3.2.2 Sequencing Artefacts and Contaminations

The current study was focused on authentic ASVs represented by identical 16S‐ITS sequences from at least two independent sequencing experiments. Additional singleton ASVs detected only in one experiment provide further insights into the composition of the cyanosphere (see below), but these sequences should be treated with caution as they could represent PCR artefacts or sporadic contaminations. However, non‐authentic sequences were rare (Text S2), and singletons did not play a major role in our datasets. The comparison of more than 40,000 established 16S‐ITS sequences from *Coleofasciculus* sp. CHI showed that 96% of the sequence counts were found among the 37 authentic ASVs validated by two sequence runs (Table S1).

#### 3.2.3 Proportion of Cyanobacterial ASVs

A comparison of the ASVs of all 32 *Coleofasciculus* cultures showed large differences in the proportion of the cyanobacterial host, ranging from less than 5% (NCR, SAH, VOE, WW12) to more than 95% (SOL, CCY0002, WIS; Figure 3). In four cultures, cyanobacterial ASVs were detected in only one of the two replicates (SAH, VOE, ASK1, ASK5). The low frequency or even absence of cyanobacterial 16S‐ITS sequences in the amplicon data of DNA from non‐axenic *Coleofasciculus* cultures provided clear evidence that the proportion of established ASVs does not reliably reflect the bacterial composition of the microbiome (see also below). This observation could be related to a different copy number of ribosomal operons in the genomes, but the more plausible explanation is a PCR bias related to primer binding, G + C content and especially the inhomogeneous amplicon length, which differs by up to 40% (1827 bp to 3044 bp). The bias could be reduced by amplifying the 16S rRNA gene instead of the complete 16S‐ITS region, but this modification would be accompanied by a significant loss of resolution.

### 3.3 Characterisation of Cyanobacteria Based on 16S‐ITS Amplicon Sequencing

#### 3.3.1 Superior Resolution Profiling of 34 Cyanobacteria (*Coleofasciculus*, *Salileptolyngbya*)

**FIGURE 4:**
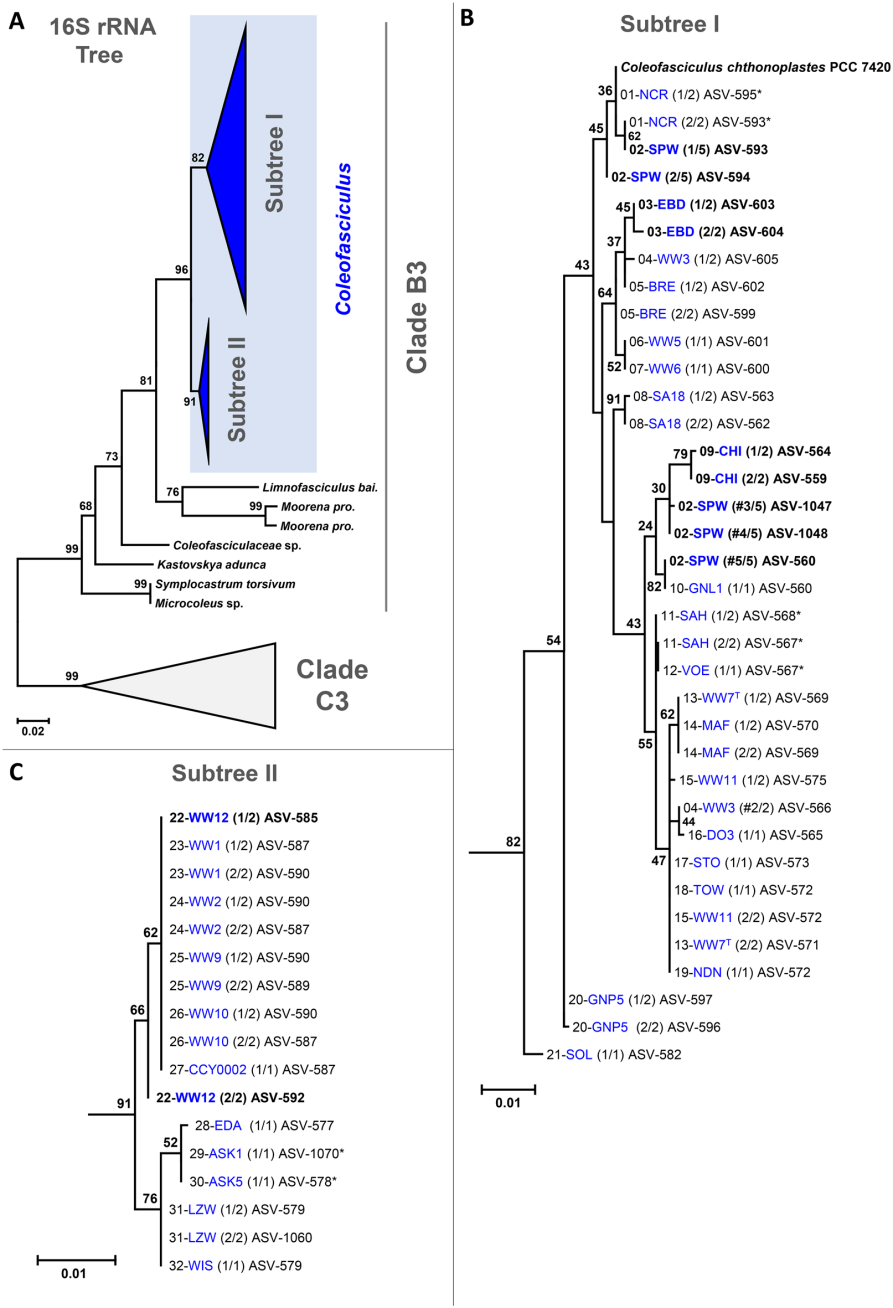
Phylogenetic Maximum Likelihood tree of 16S rDNA sequences from 32 *Coleofasciculus* strains examined in the current study. (A) Overview of the cyanobacterial phylogeny with 77 sequences from clade B3 and clade C3. The complete tree is shown in Figure S2. (B) Subtree I of newly established *Coleofasciculus* ASVs from 21 DSMZ strains (shown in blue) and the reference sequence from *Coleofasciculus* sp. PCC 7420. (C) Subtree II with 11 DSMZ strains. Strain numbering from top to bottom was used throughout the manuscript (Table 1). Genome‐sequenced strains are shown in bold. All ASVs except those marked with an asterisk were validated by two sequencing experiments (Table S1). The numbers in brackets show the number of different cyanobacterial ASVs per strain. Hashes indicate authentic secondary ASVs from probable non‐unicyanobacterial *Coleofasciculus* cultures. Polytomy in the tree without horizontal branches reflects identical 16S rRNA sequences; different ASV numbers can be attributed to sequence variations in the ITS region. ASV, amplicon sequence variant.

PacBio amplicon sequencing allowed the establishment of complete 16S‐ITS sequences from all 34 investigated cyanobacteria. The authenticity of cyanobacterial ASVs from 28 strains was confirmed by identical sequences from at least two sequencing experiments. The comparison of singleton ASVs with deposited reference sequences from strains NCR (ASV‐593, EF654039.1), SAH (ASV‐567, EF654043.1) and ASK5 (ASV‐578, EF654026.1) provided evidence that non‐validated ASVs from the six remaining strains were also free of sequencing errors. A closer look at the *Coleofasciculus* sequences and a comparison with the references available for 30 of 32 strains studied (Table S3) revealed the exceptional quality of the newly generated data set. High‐throughput sequencing allowed (i) an unambiguous determination of 27 previously unresolved nucleotide positions in eleven 16S rRNA genes, (ii) the detection of sequencing errors with SNPs and InDels in four genes, and (iii) an overall sequence improvement in terms of length due to the choice of the 23S rRNA reverse primer. Our 16S‐ITS sequences, ranging in size from 2071 to 2137 nucleotide positions (references: 1297–2022 nt; Table S3), enable profiling of very closely related *Coleofasciculus* strains even with identical 16S rRNA sequences (Figure 4). As an example, the unique 16S‐ITS sequences of strains WW5 and WW6 (ASV‐601, ASV‐600), which were isolated from the Baltic Sea (Table 1), differ only by a single InDel of 12 nucleotide positions in the ITS region. Another example is the nearly indistinguishable 16S‐ITS sequence of strains STO and TOW (ASV‐573, ASV‐572) isolated from North Sea sediment near St. Peter Ording and from pacific mangroves near Sydney, respectively, which have a single G:C SNP in the ITS region. Accordingly, the reliable determination of complete 16S‐ITS sequences without sequencing errors from cyanobacteria allows their classification at strain level and thus provides a tool for the differentiation of closely related isolates within a species.

#### 3.3.2 Detection of Ribosomal Operon Variants

**FIGURE 5:**
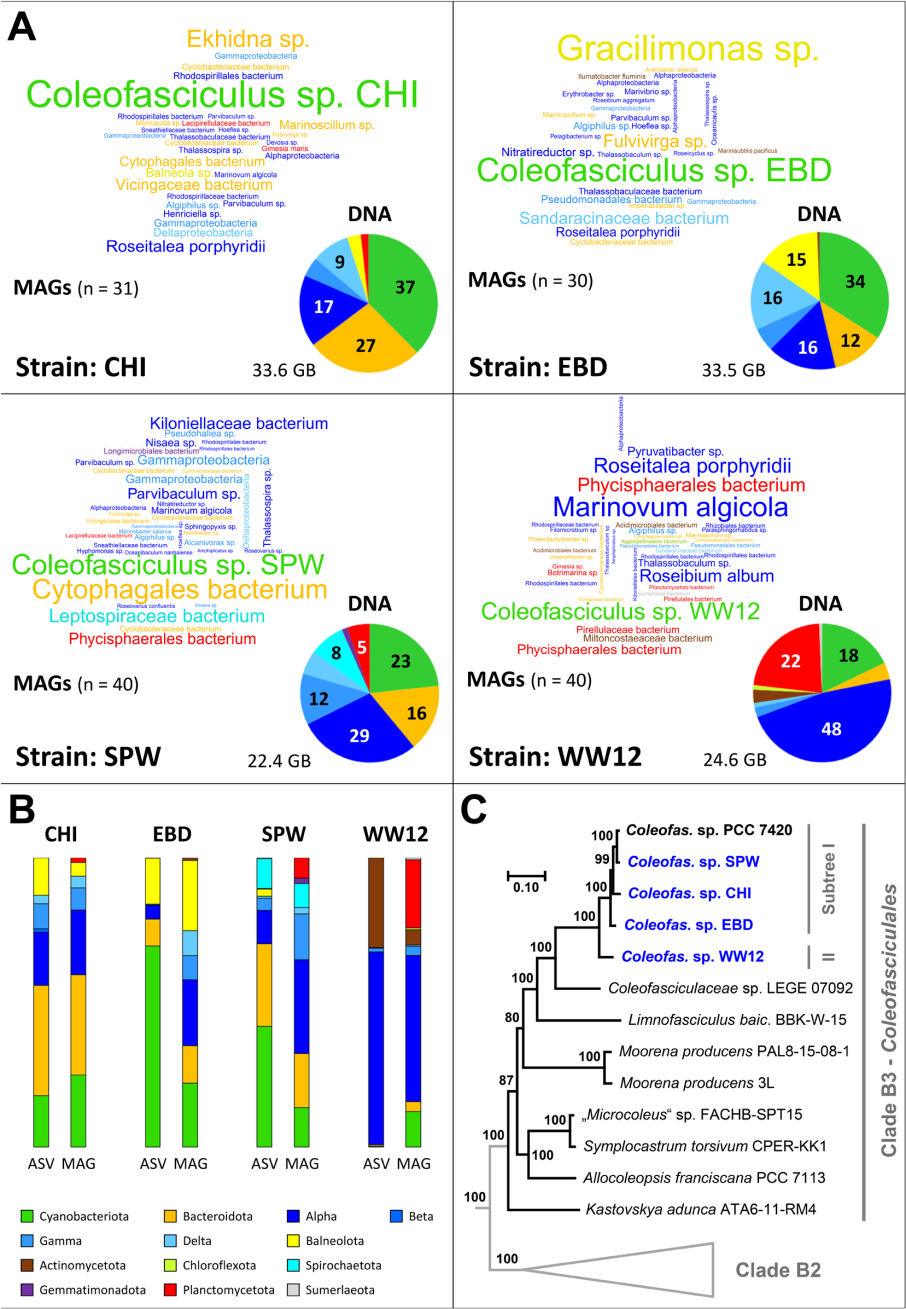
Metagenome analysis of four non‐axenic *Coleofasciculus* strains (CHI, EBD, SPW, WW12). (A) Relative abundance of MAGs in the cyanosphere. Word clouds show all high quality MAGs with completeness > 80% and a contamination rate < 10% (Table S2). Pie charts reflect their taxonomic classification on phylum level (*Pseudomonadota*: Class [*Alpha‐, Beta‐, Gamma‐, Deltaproteobacteria*]). (B) Bacterial composition of the cyanosphere based on 16S‐ITS amplicon sequencing (ASVs) and metagenomic binnning (MAGs). (C) Phylogenomic tree of five *Coleofasciculus* genomes and 37 cyanobacterial reference genomes. Newly established MAGs are highlighted in blue. The RaxML tree was constructed from 20,055 variable amino acid positions of 92 housekeeping genes under the GTR4Γ model. The complete tree is shown in Figure S3, accession numbers of reference genomes are listed in Table S8. ASVs, amplicon sequence variants; MAGs, metagenome‐assembled genomes.

20 of 34 investigated cultures contained two closely related cyanobacterial ASVs (Figures 4 and S2). They could represent different variants of the ribosomal operon in the same strain (Iteman et al. 2002; Sadeghifard et al. 2006; Arias, Olivares‐Fuster, and Goris 2010; Hakovirta et al. 2016) or incomplete purification of the isolate. The two *Coleofasciculus* ASVs in strains CHI, EBD, SPW, and WW12 were found with similar sequence counts providing strong evidence that they represent ribosomal operon variants of the same cyanobacterium (CHI: ASV‐559 [1445x], ASV‐564 [1336x]; EBD: ASV‐603 [16,057x], ASV‐604 [13,988x]; SPW: ASV‐594 [2219x], ASV‐593 [1638x]; WW12: ASV‐585 [140x], ASV‐592 [115x]; Table S1). This conclusion was independently supported by our metagenomic analyses of these four isolates, which revealed the presence of a single cyanobacterial MAG per *Coleofasciculus* culture (Figure 5A, Table S2). The detection of ribosomal operon variants in a single cyanobacterial strain hence reflects an unprecedented resolution of long‐read 16S rDNA amplicon sequencing.

#### 3.3.3 *Coleofasciculus* Isolates Containing More Than One Cyanobacterial Strain

32 cyanobacterial cultures examined belong to the genus *Coleofasciculus*, but the detection of additional ASVs with a distinct phylogenetic position indicated that only 30 of them are unicyanobacterial. The identification of three additional rare cyanobacterial ASVs in *Coleofasciculus* sp. SPW (ASV‐1047, ASV‐1048, ASV‐560) beyond the two abundant ones (ASV‐593, ASV‐594), likely shows the presence of a second *Coleofasciculus* strain in the same culture. These ASVs were also found in both sequencing runs, are distinctly located in the phylogenetic tree (Figure 4) and have a comparable but one order of magnitude lower number of about 170 versus 1900 sequence counts (Table S1). Comparably deep metagenome sequencing and binning revealed only one cyanobacterial MAG in the SPW metagenome with a 665‐fold coverage, which is lacking the 16S rRNA gene (MAG: A1‐SPW‐01, Table S2). The absence of a second, closely related *Coleofasciculus* MAG could be due to the stringent metagenomic binning pipeline with DAS‐Tool (Marter et al. 2021), which was the prerequisite for the generation of datasets with high‐quality MAGs. One should also keep in mind the general limitations of metagenomics, as assembling and binning of microbiome samples with very similar bacteria could lead to a consensus MAG. However, the two common and three rare ASVs of *Coleofasciculus* sp. SPW are only distantly related (Figure 4) and probably represent two species of this genus. Our phylogenomic analysis showed that *Coleofasciculus* sp. SPW is the closest relative of *Coleofasciculus* sp. PCC 7420 (Figures 5C and S3), and the established metagenome thus corresponds to the two abundant 16S‐ITS sequences (ASV‐593, ASV‐594; Figure 4).

In addition, the two ASVs from *Coleofasciculus* sp. WW3 (ASV‐605, ASV‐566) were also located at different positions in the phylogenetic tree, suggesting that this culture may also be comprised of two *Coleofasciculus* strains. The mixed culture hypothesis for WW3 and SPW should be confirmed experimentally in the future, either by long‐read metagenome sequencing or by manual separation and sub‐cultivation of individual filaments.

### 3.4 Phylogenetic Characterisation of the Genus *Coleofasciculus* (*Coleofasciculales*, Clade B3)

#### 3.4.1 16S rRNA Gene Phylogeny of *Coleofasciculus* ASVs

The evolutionary relationships of all 53 *Coleofasciculus* and four *Salileptolyngbya* ASVs established in the current study were analysed using 16S rRNA reference sequences from type strains and closely related genome‐sequenced cyanobacteria from clade B3 and C3, respectively (Figure S2) (Marter et al. 2021). Figure 4A shows a schematic overview of the Maximum Likelihood tree of the 16S rRNA gene that was rooted with the distinct sequences of clade C3. The common branching of all sequences of the genus *Coleofasciculus* including the type strain *C. chthonoplastes* WW7^T^ and the genome‐sequenced strain PCC 7420 is supported by 96% bootstrap proportion (BP). The formation of two distinct *Coleofasciculus* subtrees (I, II), comprising two‐thirds and one‐third of the investigated strains, respectively (Table 1), was previously reported by Siegesmund et al. (2008) based on a comparable taxon sampling. Two additionally studied strains VOE (Kattegat, Denmark) and DO3 (Baltic Sea, Germany) are grouping in subtree I (Figure 4B), and the third strain CCY0002 from the Dutch North Sea island Schiermonnikoog (Bolhuis et al. 2010) is located in subtree II (Figure 4C). The phylogenies of both studies were largely congruent, with three exceptions (see Text S3).

The phylogenetic 16S rRNA analysis illustrates the close relationship of *Coleofasciculus* strains from both subtrees. Polytomy in the tree without horizontal branches, which is exemplified for the 10 uppermost sequences of subtree II ranging from the WW12 (ASV‐585) to CCY0002 (ASV‐587; Figure 4C), reflects identical 16S rRNA genes. The presence of even indistinguishable 16S‐ITS sequences is documented for ASV‐572 of subtree I (Figure 4B), which was independently amplified from strains TOW, WW11, and NDN isolated from coastal habitats in the Pacific, Baltic Sea, and North Sea, respectively (Table 1). Further genetic differentiation of these *Coleofasciculus* strains, which showed slight differences in the width of their trichomes and cell length (Siegesmund et al. 2008), requires the establishment of complete genomes. The limitations of the 16S rRNA gene to resolve the evolutionary relationship of closely related strains are well known (Stackebrandt and Goebel 1994; Janda and Abbott 2007). The detection of 16S rRNA gene operon variants from one cyanobacterium may even exceed the phylogenetic resolution of this marker, considering a possible reciprocal loss of duplicated operons leading to paralogous genes that do not reflect the speciation event (Koonin 2005). The current study has shown that several *Coleofasciculus* strains are barely distinguishable on the basis of complete 16S‐ITS sequences, but the two operon variants of unicyanobacterial cultures are always grouping close to each other in the phylogenetic tree (Figure 4). There is therefore no indication for horizontal gene transfer of the 16S rRNA gene. The differentiation of ribosomal operon variants of the same bacterium may further help to distinguish closely related strains.

#### 3.4.2 Phylogenomic Analysis of Four Newly Established *Coleofasciculus* Genomes

The phylogenetic relationship of all five genome‐sequenced *Coleofasciculus* strains was examined with 36 reference genomes of clade B1, B2, B3, B4, B5 and clade A (Shih et al. 2013) in a phylogenomic RaxML analysis based on 92 concatenated protein sequences with 20,055 variable amino acid positions (Figure 5C). The protein tree confirmed the monophyly of clade B and the sister group relationship of clade B2 and clade B3, which represents the order *Coleofasciculales* (Strunecký, Ivanova, and Mareš 2023). The relationship of the five *Coleofasciculus* strains, which was partially unclear according to the 16S rDNA phylogeny (see above, Figure 4), was resolved by our phylogenomic analysis and supported with a BP of at least 99% (Figure 5C). *Coleofasciculus* sp. SPW is the closest relative of strain PCC 7420; it was also isolated from the Sippewisset salt marshes near Woods Hole (MA, USA) two decades after the axenic reference strain (Table 1). The Chilean strain CHI branches next, and the Mediterranean strain EBD is the most distinct genome‐sequenced isolate from subtree I, consistent with the branching pattern of the 16S rRNA gene tree. The comparably long common branch separating the taxa of subtree I from the Baltic strain WW12, the first genome‐sequenced representative of subtree II, reflects the dichotomy of the genus *Coleofasciculus* (Siegesmund et al. 2008). A comparison of the 16S rRNA genes, the ANI and digital DNA–DNA hybridization (dDDH) provided clear evidence that *Coleofasciculus* sp. WW12 represents a second species of this genus (Tables S4 and S5). Furthermore, genome‐based delineation indicated that the genus *Coleofasciculus* comprises at least four different species (Text S4).

### 3.5 Assessment of the *Coleofasciculus* Microbiome Based on 16S‐ITS Amplicon Sequencing

#### 3.5.1 General Assessment of the Bacterial Diversity in 32 *Coleofasciculus* Cultures

The composition of associated heterotrophic bacteria in the *Coleofasciculus* cultures is shown in Figure 3. Determination of the complete 16S rRNA gene enabled a reliable classification of ASVs based on the curated SILVA reference database (Quast et al. 2013), and the ITS region even allowed discrimination between different strains of a species, resulting in (for amplicon sequencing) unprecedented taxonomic resolution of the respective microbiomes. The selected primers amplified a large number of bacterial sequences (median: 55 ASVs/strain), reflecting the complex composition of the cyanosphere (Table S1). The bacterial diversity of the investigated *Coleofasciculus* cultures differed even on phylum level (*Pseudomonadota*: class level, Figure 3), which may either reflect compositional differences in their original habitat or simply more stringent previous approaches to obtain pure cyanobacterial cultures. The well‐investigated microbiome of *Coleofasciculus* sp. CHI comprises four major lineages, that is, the cyanobacterium, *Bacteroidota*, *Pseudomonadota* (*Alpha*‐, *Beta*‐, *Gamma*‐, *Deltaproteobacteria*) and *Balneolota* (Figure S1). A statistical analysis of all 610 authentic ASVs from associated bacteria detected in the 32 investigated *Coleofasciculus* microbiomes suggested that the cyanosphere of this marine genus is dominated by *Pseudomonadota* (median: 59%; *Alphaproteobacteria* [46%], *Gammaproteobacteria* [13%]), *Bacteroidota* (23%), *Actinomycetota* (6%) and *Balneolota* (6%; Table S6). The absence of *Planctomycetota* likely corresponds to individual binding sites that were not covered by the current primers (Mazzoli et al. 2020). A core set of seven commonly associated bacteria that could be classified at species level was detected in more than half of the *Coleofasciculus* cultures, namely *Balneola alkaliphila* (26/32 strains; *Balneolota*, *Balneolales*), *Nitratireductor arenosus* (26/32; *Alphaproteobacteria*, *Hyphomicrobiales*), *Roseovarius indicus* (25/32; *Alphaproteobacteria*, *Rhodobacterales*), *Roseitalea porphyridii* (23/32 strains; *Alphaproteobacteria*, *Hyphomicrobiales*), *Imperialibacter roseus* (21/32 strains; *Bacteroidota*, *Cytophagales*), *Marinovum algicola* (20/32; *Alphaproteobacteria*, *Rhodobacterales*) and *Ekhidna lutea* (17/32 strains; *Bacteroidota, Cytophagales*, Table S1). This finding offers the promising perspective to investigate the functional role of these heterotrophic bacteria for the cyanobacterial microbiome (Kost et al. 2023) and their relevance in natural microbial mats (Bolhuis, Cretoiu, and Stal 2014) by isolation of the key players and cocultivation experiments with an axenic *Coleofasciculus* strain. In addition to the dominant lineages, other authentic ASVs were detected from *Gemmatimonadota* (8×), *Chloroflexota* (5×), *Spirochaetota* (4×), *Verrucomicrobiota* (2×), *Acidobacteriota* (1×), *Chlorobiota* (1×) and *Bacillota* (1×). Overall, the current study demonstrates that 16S‐ITS PacBio amplicon sequencing is a valuable tool for the precise taxonomic assessment of bacterial diversity in cultures of non‐axenic cyanobacteria.

#### 3.5.2 The Microbiome of *Coleofasciculus* sp. CHI

16S‐ITS sequencing provided initial insights into the microbiomes of non‐axenic filamentous strains of the genus *Coleofasciculus* isolated from different coastal habitats (Figure 1; Table 1). However, the simple question of the total number of different bacterial strains living in association with a certain cyanobacterial isolate is difficult to answer, as the example of *Coleofasciculus* sp. CHI shows. It is overestimated by a total amount of 94 ASVs, which also include amplicon sequence variants, rare sequencing errors, and contaminations, but underestimated by 37 independently validated ASVs without authentic singletons (Figure 2). Accordingly, the ASV datasets of the four metagenome‐sequenced strains were manually curated to provide a more reliable overview of the heterotrophic housemates (Table S7). In the CHI dataset, we identified six heterotrophic bacteria with up to three authentic variants of the ribosomal operon, in addition to *Coleofasciculus* with two variants (ASV‐559, ASV‐564; Table S7A). Intragenomic 16S‐ITS variation was also found in four associated *Alphaproteobacteria*, namely *Thalassospira lucentensis* (ASV‐65, ASV‐66, ASV‐67; *Rhodospirillales*), *Thalassobaculum litoreum* (ASV‐137, ASV‐165; *Rhodospirillales*), *Roseovarius indicus* (ASV‐88, ASV‐91; *Rhodobacterales*), and *Parvibaculum lavamentivorans* (ASV‐112, ASV‐113; *Hyphomicrobiales*), the gammaproteobacterium *Alcanivorax venustensis* (ASV‐233, ASV‐232; *Oceanospirillales*) and *Marinoscillum* sp. as a representative of the phylum *Bacteroidota* (ASV‐412, ASV‐470; *Cytophagia*, *Cytophagales*). Our dataset of amplicon sequence variants, which was validated by at least two sequencing runs, thus revealed the presence of 28 heterotrophic bacterial species in the cyanosphere of *Coleofasciculus* sp. CHI.

Further insights into the *Coleofasciculus* microbiome of strain CHI were gained by a set of 57 unauthenticated singletons. The remarkable number of 42 unique ASVs from run BC002 (2023) correlates on the one hand with the sequencing depth, which exceeds that of the other three runs by a factor of four (25,000 versus 6000 sequence counts; Table S1). Manual curation, on the other hand, revealed the presence of several putative PCR artefacts and a massive betaproteobacterial contamination specific to this sequencing run. However, stringent filtering of PCR artefacts and contaminations resulted in 17 singletons that likely represent authentic ASVs (Figure 2; Table S7A). One of the singletons was classified as *Marinovum algicola* (ASV‐049; *Alphaproteobacteria*, *Rhodobacterales*), an intriguing marine bacterium of the Roseobacter group with flagellar and biofilm chromids that is characterised by a multipartite genome organisation (Frank et al. 2015). ASV‐049 showed 100% sequence identity with the 16S rRNA gene of the type strain *M. algicola* NBRC 16653 (NR_112651.1). *M. algicola* ASVs were identified in 20 of 32 investigated *Coleofasciculus* strains and its presence in the cyanosphere of *Coleofasciculus* sp. CHI was confirmed by metagenomic binning (D1‐CHI‐19; Table S2). Two other singletons from BC002 (2023) are corresponding to MAGs of the CHI metagenome, namely ASV‐116 from *Marinibaculum* sp. (*Alphaproteobacteria*, *Sneathiellales*; D1‐CHI‐29) and ASV‐479 from *Imperialibacter* sp. (*Bacteroidota*, *Cytophagales*; D1‐CHI‐32). Rare gammaproteobacterial 16S‐ITS singletons with species names reflecting their marine origin are represented by *Marinobacter algicola* ASV‐259 (*Alteromonadales*) and *Woeseia oceani* ASV‐269 (*Chromatiales*).

Overall, 16S‐ITS amplicon sequencing indicated that at least 45 heterotrophic bacteria (17 singletons, 28 validated ASVs) are living in the cyanosphere of *Coleofasciculus* sp. CHI (= DSM 104232). Metagenome sequencing detected two additional MAGs from *Planctomycetota* (D1‐CHI‐18, D1‐CHI‐24) and one MAG from *Bacteroidota* (D1‐CHI‐04; Table S2A), which were not represented by the ASVs. Accordingly, a total number of 48 associated bacteria, representing *Pseudomonadota* (31×), *Bacteroidota* (12×), *Actinomycetota* (2×), *Planctomycetota* (2×) and *Balneolota* (1×), are essentially living from exudates of the cyanobacterium. The marine microbiome of the filamentous CHI culture (Figure 1), which was sampled in 1994 from the intertidal zone at the Dichato Marine Station in Chile (Table 1; (Karsten 1996)) and has been continuously cultivated for three decades, thus still exhibits a striking complexity, even exceeding that of the recently characterised non‐axenic limnic cyanobacterium *Stigonema ocellatum* DSM 106950 for which 27 associated heterotrophic bacteria were reported (Marter et al. 2021).

### 3.6 Comparison of Metagenome and 16S‐ITS Amplicon Data of Four *Coleofasciculus* Strains

#### 3.6.1 Benefits and Limitations of Metagenome Versus Amplicon Sequencing

Metagenome sequencing and binning of *Coleofasciculus* sp. CHI resulted in 30 high‐quality MAGs of heterotrophic bacteria (Figure 5A; Table S2A). A comparison between metagenome and 16S‐ITS amplicon sequencing showed that most MAGs are represented by corresponding ASVs. However, the discovery of *Planctomycetota* MAGs in three *Coleofasciculus* metagenomes is remarkable (Figure 5; Table S2), as they were absent from our 16S‐ITS amplicon data. In contrast, our manually curated set of ASVs from *Coleofasciculus* sp. CHI indicates the presence of 17 additional heterotrophic bacteria that were not found in the corresponding metagenome (Table S7A). Particularly noteworthy is the absence of the commonly associated species *Nitratoreductor arenosus* (ASV‐130) and *Roseovarius indicus* (ASV‐88, ASV‐91). Future studies based on long‐read metagenome sequencing could answer the question if the missing MAGs are the result of incomplete binning or an insufficient sequencing depth.

The absence of a second cyanobacterial MAG in the metagenome of SPW (665‐fold coverage), which represents a mixed *Coleofasciculus* culture of two or three different strains (Figure 4), suggests that the dominant cyanobacterium is at least two orders of magnitude more abundant than the co‐inhabitant(s). It is worth noting that metagenome sequencing and binning failed to detect a second *Coleofasciculus* species despite the remarkable sequencing depth. High‐throughput amplicon sequencing of the 16S rRNA gene allowed a much deeper resolution based on 12,000 sequence counts (Table S1) and is therefore the method of choice for ‘contaminant’ detection or confirmation of the unicyanobacterial status of a culture.

#### 3.6.2 Metagenome‐Assembled Genomes and the Absence of the 16S rRNA Gene

Metagenome assembly and binning of the four investigated non‐axenic *Coleofasciculus* strains exemplified the limitations of short‐read sequencing with respect to the 16S rRNA gene. The MAGs of CHI, SPW, and WW12 did not contain any 16S rRNA sequences, regardless of the calculated genome completeness between 95% and 99% (Table S2). The absence of the 16S rDNA in MAGs of non‐axenic cyanobacteria reflects typical binning problems due to a deviant nucleotide composition and multiple copy numbers of the ribosomal operon (Roller, Stoddard, and Schmidt 2016; Marter et al. 2021). With respect to the most important marker gene of microbial taxonomy, the 16S rRNA (Stackebrandt and Ebers 2006; Yarza et al. 2014), amplicon sequencing of the complete 16S‐ITS region allows to overcome the limitations of short‐read metagenome sequencing.

#### 3.6.3 Comparison of Quantitative Metagenome and Amplicon Data

The metagenomic binning results from four *Coleofasciculus* cultures (CHI, EBD, SPW, WW12) served as a reference for the quantitative assessment of our 16S‐ITS data. However, a comparison of ASV and metagenome classification on phylum level revealed major quantitative differences in the composition of the ASVs, which propose that the 16S‐ITS amplicon data do not reliably reflect the proportion of bacteria in the cyanosphere (Text S5). Accordingly, PacBio‐based 16S‐ITS amplicon sequencing is a valuable tool for (i) retrieving complete cyanobacterial sequences from non‐axenic strains and (ii) analysing the composition of medium‐ and low‐complexity communities, but the ASVs should not be used for quantification purposes.

### 3.7 Bacterial Isolates From *Coleofasciculus* sp. WW12 in the Light of 16‐ITS Amplicon and Metagenome Sequencing

*Coleofasciculus* sp. WW12 exhibited the most diverse microbiome of the current study with 75 ASVs from different bacterial strains (excluding rRNA operon variants) and 40 high‐quality MAGs (Tables S2D and S7‐04) and was therefore selected for an isolation experiment. The isolation of 15 different heterotrophic bacterial species from its cyanosphere allowed a comparison between cultivation‐dependent and cultivation‐independent approaches (Table 2). We were able to isolate the six most common bacteria detected in more than half of the 32 investigated *Coleofasciculus* cultures (see above), namely *Balneola alkaliphila* (26/32), *Nitratireductor arenosus* (26/32), *Roseovarius indicus* (25/32), *Roseitalea porphyridii* (23/32), *Imperialibacter roseus* (21/32) and *Marinovum algicola* (20/32). The majority of isolates were comparably well‐growing *Alphaproteobacteria* (7× *Hyphomicrobiales*, 2× *Rhodobacterales*, 1× *Sphingomonadales*), as expected based on the chosen marine broth medium. It is noteworthy that only five of 15 isolates could be detected in the respective microbiome based on metagenome binning. In particular, the absence of *B. alkaliphila* and *R. indicus* MAGs, which (i) were the most common and third most common bacteria in the cyanosphere of *Coleofasciculus* and (ii) were represented by 5722 and 1801 amplicon sequences, respectively (Table 2), likely reflects incomplete binning with the stringent DAS tool pipeline (Sieber et al. 2018). Missing MAGs of the other seven isolates, including *Stappia indica* , which was only detected by eight of 74,763 ASV counts (4× BC006 [2019], 4× BC007 [2023]; Table S7D), probably reflect a still insufficient sequencing depth, notwithstanding the establishment of 24.6 GB of sequence data from the microbiome of *Coleofasciculus* sp. WW12 (Figure 5A). However, 16S‐ITS amplicon sequencing confirmed the authenticity of all 15 isolates. Comparable results were recently obtained for the marine alga *Chromera velia*, which also yielded metagenomic fingerprints for only six of 16 isolated bacteria (Koteska et al. 2023). Accordingly, the newly established 16S‐ITS amplicon sequencing approach is a very promising tool for the reliable detection and classification of heterotrophic bacteria from non‐axenic algae, protists and cell cultures.

**TABLE 2:** List of 15 bacteria isolated from the cyanosphere of *Coleofasciculus* sp. WW12 (= DSM 104231).

		Classification			Amplicon sequencing				Metagenome sequencing	
	Isolate	Genus‐species	Phylum (class)	Order	ASV	ASV/BC	Counts	Occurrence	MAG	Coverage
01	WW12_LAS_4	*Balneola alkaliphila*	*Balneolota*	*Balneolales*	**ASV‐320** ^**a**^	4/4	5722	1st (26/32)	No MAG	x
02	WW12_G5_13	*Roseitalea porphyridii*	*Alphaproteobacteria*	*Hyphomicrobiales*	**ASV‐152**	4/4	4856	4th (23/32)	**G1‐WW12‐03**	512
03	WW12_11_A1	*Nitratireductor arenosus*	*Alphaproteobacteria*	*Hyphomicrobiales*	**ASV‐130**	4/4	48	2nd (26/32)	No MAG	x
04	WW12_P6‐3A	*Marinovum algicola*	*Alphaproteobacteria*	*Rhodobacterales*	**ASV‐048**	2/4	270	6th (20/32)	**G1‐WW12‐01**	715
05	WW12_H3	*Roseibium album*	*Alphaproteobacteria*	*Hyphomicrobiales*	**ASV‐074**	2/4	106	(10/32)	**G1‐WW12‐05**	404
06	WW12_P6D	*Parasphingorhabdus* sp.	*Alphaproteobacteria*	*Sphingomonadales*	**ASV‐185**	2/4	96	(5/32)	**G1‐WW12‐16**	54
07	WW12_P2_4	*Roseovarius indicus*	*Alphaproteobacteria*	*Rhodobacterales*	**ASV‐013**	2/4	1801	3rd (25/32)	No MAG	x
08	WW12_G5_7	*Roseibium aggregatum*	*Alphaproteobacteria*	*Hyphomicrobiales*	**ASV‐664**	2/4	355	(13/32)	No MAG	x
09	WW12_1C	*Acuticoccus* sp.	*Alphaproteobacteria*	*Hyphomicrobiales*	**ASV‐127**	4/4	34	(2/32)	No MAG	x
10	WW12_M2	*Devosia marina*	*Alphaproteobacteria*	*Hyphomicrobiales*	**ASV‐120**	3/4	31	(11/32)	No MAG	x
11	WW12_G1_3	*Chryseoglobus indicus*	*Actinomycetota*	*Micrococcales*	**ASV‐360**	2/4	29	(13/32)	No MAG	x
12	WW12_A1_3	*Imperialibacter roseus*	*Bacteroidota*	*Cytophagales*	**ASV‐481**	2/4	16	5th (21/32)	**G1‐WW12‐35**	12
13	WW12_B	*Pacificispira* sp.	*Alphaproteobacteria*	*Rhodospirillales*	**ASV‐722**	1/4	13	(10/32)	No MAG	x
14	WW12_P14	*Stappia indica*	*Alphaproteobacteria*	*Hyphomicrobiales*	**ASV‐117**	2/4	8	(10/32)	No MAG	x
15	WW12_LAS_3	*Marinoscillum luteum*	*Bacteroidota*	*Cytophagales*	**ASV‐443**	2/4	7	(16/32)	No MAG	x

*Note:* Isolates with corresponding metagenome‐assembled genomes (MAGs) were classified with GTDB‐Tk, all other isolates with SILVA based on their 16S rRNA sequence. Further information about the curated amplicon sequence variants (ASVs) is provided in Table S7‐04 (SAMN6736235), corresponding information from metagenomic binning is shown in Table S2D (SRR26147846). ASVs and MAGs that are corresponding to specific isolates are highlighted in blue; missing MAGs are shown in red. 16S rRNA gene sequences of the isolates are provided in Table S7‐04.

Abbreviation: BC, barcode.

^a^ *Balneola alkaliphila* is represented by two ASVs (ASV‐320, ASV‐322).

## 4 Conclusions

In the last decade, metagenomics has provided extensive insights into the hidden world of uncultured *Bacteria* and *Archaea*, referred to as the ‘microbial dark matter’ (Rinke et al. 2013; Hug et al. 2016). Genome‐sequencing is meanwhile mandatory for the description of new species and the bacterial taxonomy was substantially improved by phylogenomic analyses (Meier‐Kolthoff et al. 2022; Parks et al. 2022). So one could ask heretically: Do we still need the 16S rRNA at all? And the answer is: Yes! The current study has clearly documented that the 16S rRNA gene is a very important diagnostic marker even in the post‐genomic era. 16S‐ITS amplicon sequencing resulted in a sequencing depth that exceeds that of metagenome sequencing by several orders of magnitude and likely enables the detection of blind spots in metagenome binning. The absence of ASVs from *Planctomycetota* due to the selected primer set was in turn complemented by the corresponding MAGs. A combined approach of 16S‐ITS amplicon and metagenome sequencing is currently the method of choice to investigate non‐axenic cyanobacteria with a microbiome of ‘medium complexity’ (20–100 taxa), while deep metagenome sequencing alone might be sufficient for a comprehensive analysis of ‘low complexity’ consortia with less than 20 heterotrophs in the cyanosphere.

The current study exemplified that high‐throughput amplicon sequencing of the complete 16S‐ITS region allows a rapid taxonomic assessment of morphologically indistinguishable cyanobacteria. The established pipeline would be suitable for high‐throughput screening of non‐axenic strains with an automated taxonomic analysis. Sequencing of a median number of more than 10,000 high‐quality 16S‐ITS sequences per barcode provided first insights into the diversity of associated heterotrophic bacteria in the cyanosphere of 32 *Coleofasciculus* strains. Long‐read PacBio sequencing of the nearly complete 16S‐ITS generally results in a much better taxonomic resolution than short‐read Illumina sequencing. The reasonable sequencing depth in the current study confirmed that most of the strains were unicyanobacterial (30/32), but the presence of additional very closely related cyanobacteria in the *Coleofasciculus* cultures SPW and WW3 would have been overlooked based on metagenome sequencing and binning alone. Furthermore, a well‐known weakness of metagenomic binning, namely the absence of the 16S‐rRNA gene in many MAGs (Cornet et al. 2018; Marter et al. 2021), could be compensated by 16S‐ITS sequencing. In the near future, these technical problems may be solved by long‐read metagenomics, leading to complete genomes. Nevertheless, the current study showed that the sequencing depth is a limiting factor in metagenomics. For simple diagnostic purposes, the costs of amplicon sequencing per base pair are more than three orders of magnitude lower compared to (meta‐)genome sequencing (16S‐ITS: ∼2,300 bp; genome: ∼4 GB). Accordingly, amplicon sequencing of the 16S rRNA gene will still be an extremely valuable tool for one of the most challenging tasks in cyanobacterial and algal microbiology—the rapid characterisation of non‐axenic strains.

## Author Contributions

**Pia Marter:** investigation, formal analysis, data curation, writing – original draft, methodology, validation. **Heike M. Freese:** writing – original draft, methodology, software, formal analysis, data curation, validation. **Victoria Ringel:** investigation, writing – review and editing, formal analysis, validation. **Henner Brinkmann:** methodology, formal analysis, writing – review and editing, data curation, investigation. **Silke Pradella:** writing – review and editing, data curation, resources. **Manfred Rohde:** methodology, visualization, formal analysis, investigation, resources. **Michal Jarek:** formal analysis, methodology, investigation, resources. **Cathrin Spröer:** investigation, methodology, formal analysis, resources. **Irene Wagner‐Döbler:** writing – review and editing. **Jörg Overmann:** writing – review and editing, conceptualization, resources. **Boyke Bunk:** formal analysis, methodology, resources. **Jörn Petersen:** conceptualization, investigation, funding acquisition, writing – original draft, validation, visualization, writing – review and editing, formal analysis, project administration, data curation, supervision, methodology.

## Ethics Statement

The authors have nothing to report.

## Conflicts of Interest

The authors declare no conflicts of interest.

## Supporting information


**Figure S1.** Bacterial composition of 32 non‐axenic *Coleofasciculus* strains based on 16S‐ITS amplicon sequencing. The bar graph shows the ASVs from all sequencing runs (Table S1). ASVs, amplicon sequence variants.

**Figure S2.** Phylogenetic Maximum Likelihood tree of 77 cyanobacterial 16S rDNA sequences. Newly established amplicon sequence variants (ASVs) from 32 *Coleofasciculus* and two *Salileptolyngbya* strains are highlighted in blue. Hashmarks indicate authentic secondary ASVs from probable non‐unicyanobacterial *Coleofasciculus* cultures. The ML tree was constructed from 1428 nucleotide positions based on the Kimura 2‐parameter model and rooted with clade C3. The tree with the highest log likelihood (−8028.89) is shown, a discrete Gamma distribution was used to model evolutionary rate differences among sites (five categories [+G, parameter = 0.1830]). The tree is drawn to scale, with branch lengths measured in the number of substitutions per site. All ASVs except those that are marked by an asterisk were validated by two sequencing experiments. Genome‐sequenced strains are shown in bold. Accession numbers of reference sequences are shown in brackets, ASV sequences from the current study are presented in Table S1.

**Figure S3.** Phylogenomic tree of four newly established *Coleofasciculus* MAGs (highlighted in blue) and 37 cyanobacterial reference genomes from clade B1, B2, B3 B4, B5 and clade A. The RaxML tree was constructed from 20,055 variable amino acid positions of 92 housekeeping genes under the GTR4Γ model and rooted with clade A. *G. herdmannii*, *Geminocystis herdmanii*; Accession numbers of reference genomes are listed in Table S8.

**Table S1A.** Primer barcodes (BCs) for PCR amplification of the 16S rRNA gene and the internal transcribed spacer (ITS) region.
**Table S1B.** Amplicon sequence variants of the 16S‐ITS region from 34 non‐axenic cyanobacteria of the genera Coleofasciculus and Salileptolyngbya. Amplicon sequencing was performed in up to four replicates with different barcodes (blue, red, grey, yellow; see Table S1A) from DNA sampled either in 2019 or 2023. Cyanobacterial ASVs are highlighted in blue.

**Table S2A.** Binning, quality assessment and taxonomic classification of the metagenome of *Coleofasciculus* sp. CHI—DSM 104232 [JP188; SRR26209547]. MAGs are ordered according to their coverage. Low quality MAGs with a completeness < 80% or a contamination rate > 10% are shown in grey. Cyanobacterial MAGs are highlighted in green.
**Table S2B.** Binning, quality assessment and taxonomic classification of the metagenome of *Coleofasciculus* sp. EBD—DSM 104233 [JP191; SRR26209553]. MAGs are ordered according to their coverage. Low quality MAGs with a completeness < 80% or a contamination rate > 10% are shown in grey. Cyanobacterial MAGs are highlighted in green.
**Table S2C.** Binning, quality assessment and taxonomic classification of the metagenome of *Coleofasciculus* sp. SPW—DSM 104237 [JP190; SRR26209612]. MAGs are ordered according to their coverage. Low quality MAGs with a completeness < 80% or a contamination rate > 10% are shown in grey. Cyanobacterial MAGs are highlighted in green.
**Table S2D.** Binning, quality assessment and taxonomic classification of the metagenome of *Coleofasciculus* sp. WW12—DSM 104231 [JP189; SRR26147846]. MAGs are ordered according to their coverage. Low quality MAGs with a completeness < 80% or a contamination rate > 10% are shown in grey. Cyanobacterial MAGs are highlighted in green.

**Table S3.** Comparison of authentic 16S‐ITS amplicon sequence variants (ASVs) of 32 *Coleofasciculus* strains from the current study with reference sequences deposited at the NCBI.

**Table S4.** Comparison of the 16S rRNA gene from 16S‐ITS amplicon sequence variants (ASVs) of 34 cyanobacteria investigated in the current study (*Coleofasciculus, Salileptolyngbya*) and eight reference strains. 16S rRNA gene identities of *Coleofasciculus* sp. SOL and other Coleofasciculus strains of subtree I below 98.7% are highlighted in bold.

**Table S5.** dDDH and ANI genome distance matrix of five Coleofasciculus genomes (dDDH: Formula 2 [blue]; OrthoANIu [red]).

**Table S6.** Percentage of non‐cyanobacterial 16S‐ITS ASVs per Coleofasciculus culture. The data set was restricted to authentic ASVs that were independently detected in at least two replicates (Table S1). Min/Max, minimal and maximal proportion per culture; Q1, 25th percentile; Median, 50th percentile; Q3, 75th percentile; SD, standard deviation. Classification on phylum level (*Pseudomonadota*: class). Lineages with a median proportion > 5% are highlighted in blue. Box plots were generated with ggplot2 (3.5.1 [Wickham 2016]) and show median, 25% and 75% percentiles, whiskers represents the 1.5*interquartile range and black dots outliers. The grey dots represent the values of each culture.

**Table S7.** Curated dataset of amplicon sequence variants of the 16S‐ITS region from *Coleofasciculus* strains CHI, EBD, SPW and WW12. The data were retrieved from the uncurated Table S1B. Metagenome‐assembled genomes (MAGs; Table S2) that are corresponding to specific ASVs are shown in bold and blue (column D). The absence of MAGs corresponding to a specific ASV is highlighted in red with “no MAG.”

**Table S8.** Reference genomes for the phylogenomic analysis of four ***Coleofasciculus*** MAGs (Figures 5C and S3).

**Text S1.** Effect of primer barcodes and DNA sampling on 16S‐ITS amplicon sequencing.
**Text S2**. Sequencing artefacts and contaminations.
**Text S3**. Comparison of 16S rRNA gene phylogenies.
**Text S4**. Genome comparison with digital DNA–DNA hybridization (dDDH).
**Text S5**. Comparison of quantitative metagenome and amplicon data.

## Data Availability

All 34 cyanobacteria examined in the current study were taken from the public collection of the DSMZ. Sequence data from all cultures were deposited at the NCBI database in 34 BioProjects (Table 1). Raw data from 16S‐ITS amplicon sequencing were deposited as individual BioSamples in the sequence read archive (SRA; see Table S1). All authentic amplicon sequence variants (ASVs) of the 16S rRNA gene and the adjacent ITS region were deposited at the NCBI repository (OR267348, OR267349, OR297700—OR297946, OR335894—OR336048, OR339247—OR339535); a complete list of all ASVs with the corresponding nucleotide sequences is presented in Table S1. Raw data from metagenome sequencing were deposited as individual BioSamples in the sequence read archive (*Coleofasciculus* sp. DSM 104232 [CHI]: SRR26209547; *Coleofasciculus* sp. DSM 104233 [EBD]: SRR26209553; *Coleofasciculus* sp. DSM 104237 [SPW]: SRR26209612; *Coleofasciculus* sp. DSM 104231 [WW12]: SRR26147846). All automatically annotated MAGs of the current study were deposited at the NCBI repository (*Coleofasciculus* sp. DSM 104232 [CHI]: SAMN36814870—SAMN36814901; *Coleofasciculus* sp. DSM 104233 [EBD]: SAMN36814729—SAMN36814762; *Coleofasciculus* sp. DSM 104237 [SPW]: SAMN36798168—SAMN36798213; *Coleofasciculus* sp. DSM 104231 [WW12]: SAMN36815369—SAMN36815413).
