# Supplementary material for: Superior Resolution Profiling of the Coleofasciculus Microbiome by Amplicon Sequencing of the Complete 16S rRNA Gene and ITS Region
Source: Environ Microbiol Rep. 2025 Jan 31;17(1):e70066. doi: 10.1111/1758-2229.70066 (PMC11785472; doi:10.1111/1758-2229.70066)
Supplement: Supplementary file 1 — Figure S1. Bacterial composition of 32 non‐axenic Coleofasciculus strains based on 16S‐ITS amplicon sequencing. The bar graph shows the ASVs from all sequencing runs (Table S1). ASVs, amplicon sequence variants. [file EMI4-17-e70066-s003.pptx]

## Slide 1
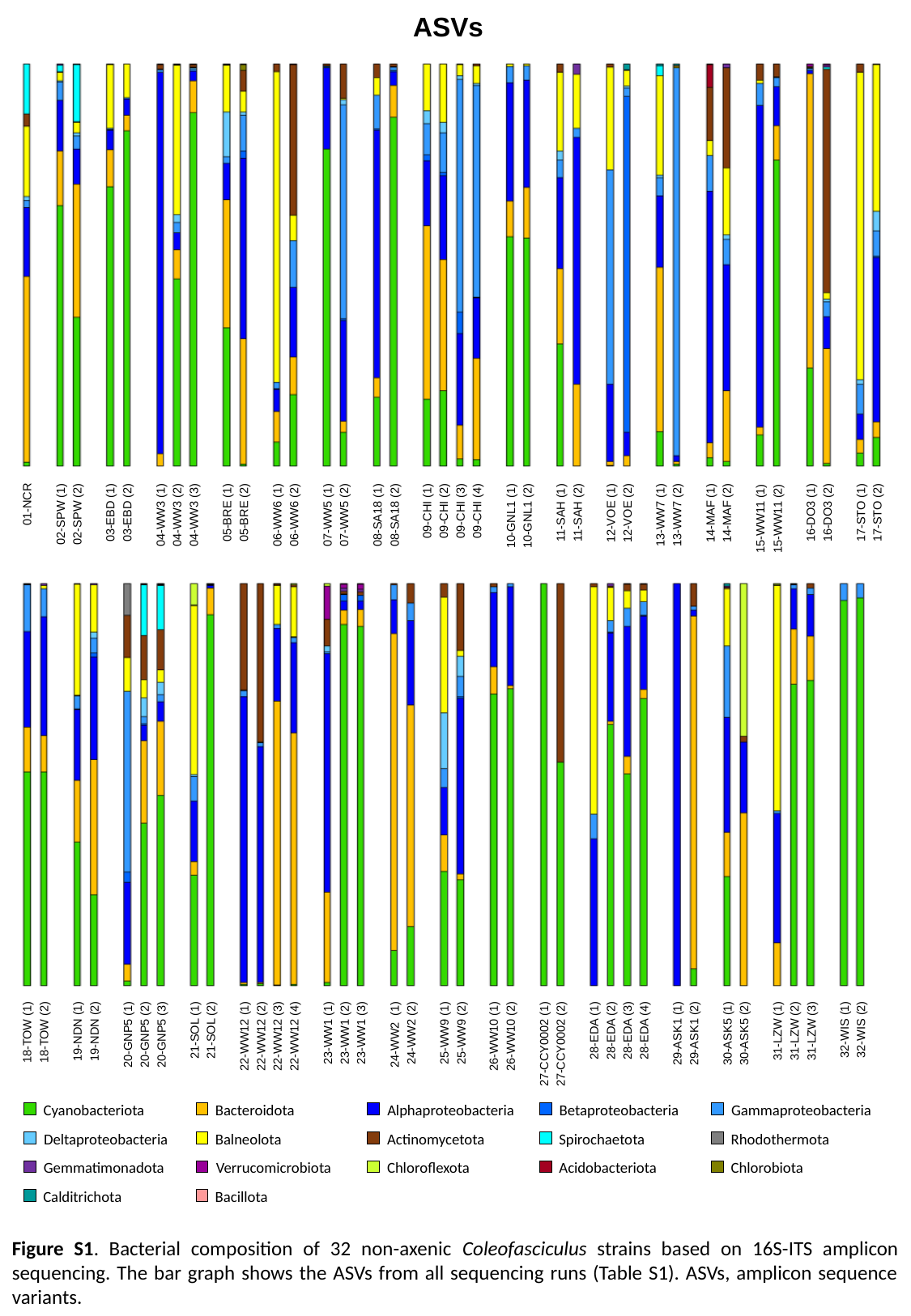

ASVs
 01-NCR
 09-CHI (1)
 09-CHI (2)
 09-CHI (3)
 09-CHI (4)
 03-EBD (1)
 03-EBD (2)
 05-BRE (1)
 05-BRE (2)
 11-SAH (1)
 11-SAH (2)
 16-DO3 (1)
 16-DO3 (2)
 17-STO (1)
 17-STO (2)
 12-VOE (1)
 12-VOE (2)
 14-MAF (1)
 14-MAF (2)
 02-SPW (1)
 02-SPW (2)
 08-SA18 (1)
 08-SA18 (2)
 04-WW3 (1)
 04-WW3 (2)
 04-WW3 (3)
 06-WW6 (1)
 06-WW6 (2)
 07-WW5 (1)
 07-WW5 (2)
 13-WW7 (1)
 13-WW7 (2)
 10-GNL1 (1)
 10-GNL1 (2)
 15-WW11 (1)
 15-WW11 (2)
 32-WIS (1)
 32-WIS (2)
 21-SOL (1)
 21-SOL (2)
 28-EDA (1)
 28-EDA (2)
 28-EDA (3)
 28-EDA (4)
 31-LZW (1)
 31-LZW (2)
 31-LZW (3)
 19-NDN (1)
 19-NDN (2)
 18-TOW (1)
 18-TOW (2)
 23-WW1 (1)
 23-WW1 (2)
 23-WW1 (3)
 24-WW2 (2)
 25-WW9 (1)
 25-WW9 (2)
 29-ASK1 (1)
 29-ASK1 (2)
 30-ASK5 (1)
 30-ASK5 (2)
 20-GNP5 (1)
 20-GNP5 (2)
 20-GNP5 (3)
 24-WW2 (1)
 22-WW12 (1)
 22-WW12 (2)
 22-WW12 (3)
 22-WW12 (4)
 26-WW10 (1)
 26-WW10 (2)
 27-CCY0002 (1)
 27-CCY0002 (2)
Cyanobacteriota
Bacteroidota
Alphaproteobacteria
Betaproteobacteria
Gammaproteobacteria
Deltaproteobacteria
Balneolota
Actinomycetota
Spirochaetota
Rhodothermota
Gemmatimonadota
Verrucomicrobiota
Chloroflexota
Acidobacteriota
Chlorobiota
Calditrichota
Bacillota
Figure S1. Bacterial composition of 32 non-axenic Coleofasciculus strains based on 16S-ITS amplicon sequencing. The bar graph shows the ASVs from all sequencing runs (Table S1). ASVs, amplicon sequence variants.
