# Supplementary material for: Superior Resolution Profiling of the Coleofasciculus Microbiome by Amplicon Sequencing of the Complete 16S rRNA Gene and ITS Region
Source: Environ Microbiol Rep. 2025 Jan 31;17(1):e70066. doi: 10.1111/1758-2229.70066 (PMC11785472; doi:10.1111/1758-2229.70066)
Supplement: Supplementary file 2 — Figure S2. Phylogenetic Maximum Likelihood tree of 77 cyanobacterial 16S rDNA sequences. Newly established amplicon sequence variants (ASVs) from 32 Coleofasciculus and two Salileptolyngbya strains are highlighted in blue. Hashmarks indicate authentic secondary ASVs from probable non‐unicyanobacterial Coleofasciculus cultures. The ML tree was constructed from 1428 nucleotide positions based on the Kimura 2‐parameter model and rooted with clade C3. The tree with the highest log likelihood (−8028.89) is shown, a discrete Gamma distribution was used to model evolutionary rate differences among sites (five categories [+G, parameter = 0.1830]). The tree is drawn to scale, with branch lengths measured in the number of substitutions per site. All ASVs except those that are marked by an asterisk were validated by two sequencing experiments. Genome‐sequenced strains are shown in bold. Accession numbers of reference sequences are shown in brackets, ASV sequences from the current study are presented in Table S1. [file EMI4-17-e70066-s010.pptx]

## Slide 1
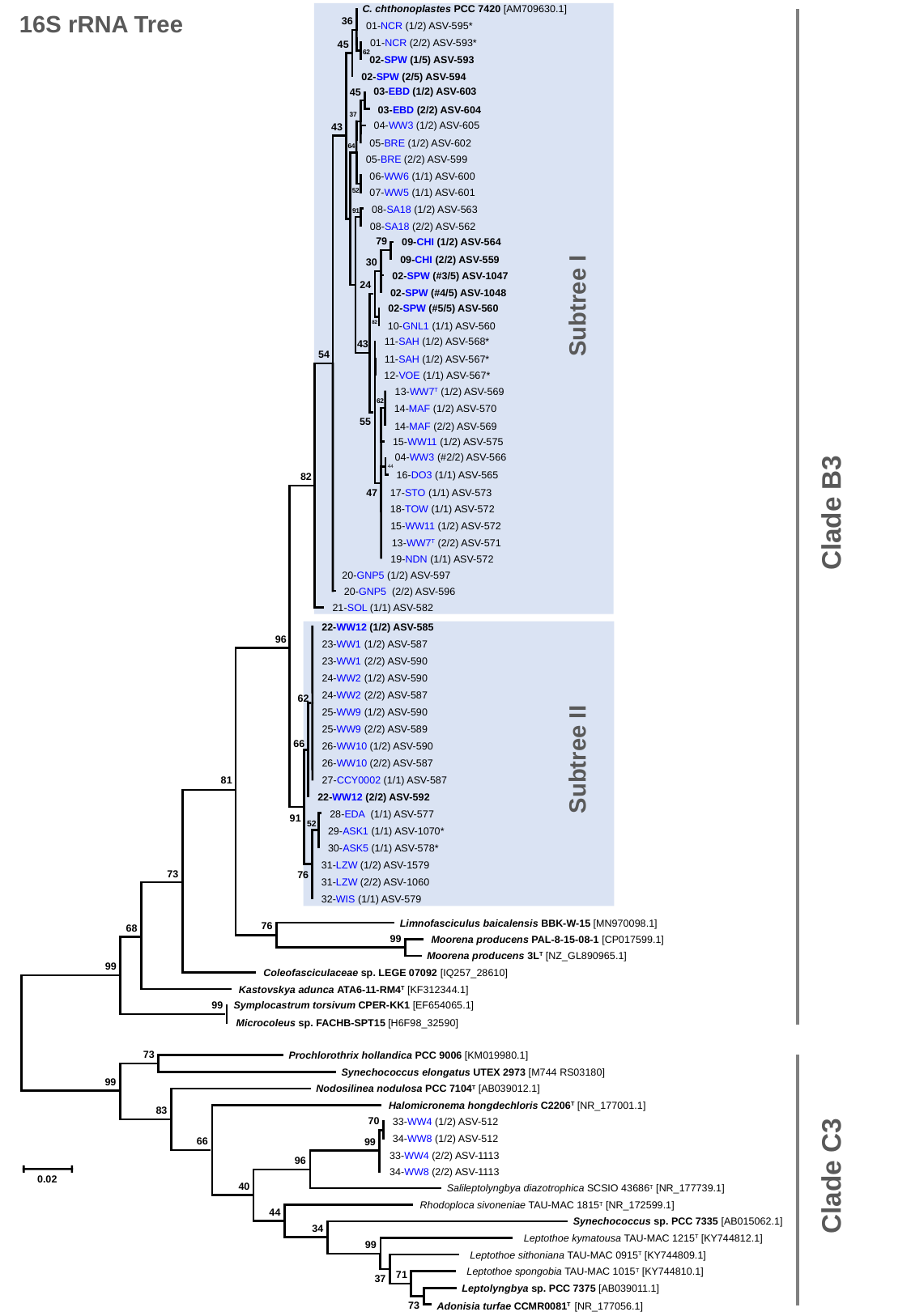

C. chthonoplastes PCC 7420 [AM709630.1]
 16S rRNA Tree
36
45
62
45
37
43
64
52
91
79
30
24
82
43
54
62
55
44
82
47
 01-NCR (1/2) ASV-595*
 01-NCR (2/2) ASV-593*
 02-SPW (1/5) ASV-593
 02-SPW (2/5) ASV-594
 03-EBD (1/2) ASV-603
 03-EBD (2/2) ASV-604
 04-WW3 (1/2) ASV-605
 05-BRE (1/2) ASV-602
 05-BRE (2/2) ASV-599
 06-WW6 (1/1) ASV-600
 07-WW5 (1/1) ASV-601
 08-SA18 (1/2) ASV-563
 08-SA18 (2/2) ASV-562
 09-CHI (1/2) ASV-564
 09-CHI (2/2) ASV-559
 02-SPW (#3/5) ASV-1047
 02-SPW (#4/5) ASV-1048
 Subtree I
 02-SPW (#5/5) ASV-560
 10-GNL1 (1/1) ASV-560
 11-SAH (1/2) ASV-568*
 11-SAH (1/2) ASV-567*
 12-VOE (1/1) ASV-567*
 13-WW7T (1/2) ASV-569
 14-MAF (1/2) ASV-570
 14-MAF (2/2) ASV-569
 15-WW11 (1/2) ASV-575
 04-WW3 (#2/2) ASV-566
 16-DO3 (1/1) ASV-565
 17-STO (1/1) ASV-573
 Clade B3
 18-TOW (1/1) ASV-572
 15-WW11 (1/2) ASV-572
 13-WW7T (2/2) ASV-571
 19-NDN (1/1) ASV-572
 20-GNP5 (1/2) ASV-597
 20-GNP5 (2/2) ASV-596
 21-SOL (1/1) ASV-582
 22-WW12 (1/2) ASV-585
 23-WW1 (1/2) ASV-587
 23-WW1 (2/2) ASV-590
 24-WW2 (1/2) ASV-590
 24-WW2 (2/2) ASV-587
 25-WW9 (1/2) ASV-590
 25-WW9 (2/2) ASV-589
 26-WW10 (1/2) ASV-590
 26-WW10 (2/2) ASV-587
 27-CCY0002 (1/1) ASV-587
 22-WW12 (2/2) ASV-592
 28-EDA (1/1) ASV-577
 29-ASK1 (1/1) ASV-1070*
 30-ASK5 (1/1) ASV-578*
 31-LZW (1/2) ASV-1579
 31-LZW (2/2) ASV-1060
 32-WIS (1/1) ASV-579
96
62
66
91
52
76
 Subtree II
81
73
Limnofasciculus baicalensis BBK-W-15 [MN970098.1]
76
68
99
 Moorena producens PAL-8-15-08-1 [CP017599.1]
Moorena producens 3LT [NZ_GL890965.1]
99
 Coleofasciculaceae sp. LEGE 07092 [IQ257_28610]
Kastovskya adunca ATA6-11-RM4T [KF312344.1]
99
Symplocastrum torsivum CPER-KK1 [EF654065.1]
 Microcoleus sp. FACHB-SPT15 [H6F98_32590]
73
Prochlorothrix hollandica PCC 9006 [KM019980.1]
Synechococcus elongatus UTEX 2973 [M744 RS03180]
99
Nodosilinea nodulosa PCC 7104T [AB039012.1]
Halomicronema hongdechloris C2206T [NR_177001.1]
83
70
 33-WW4 (1/2) ASV-512
 34-WW8 (1/2) ASV-512
66
99
 33-WW4 (2/2) ASV-1113
96
 Clade C3
 34-WW8 (2/2) ASV-1113
0.02
40
Salileptolyngbya diazotrophica SCSIO 43686T [NR_177739.1]
Rhodoploca sivoneniae TAU-MAC 1815T [NR_172599.1]
44
Synechococcus sp. PCC 7335 [AB015062.1]
34
Leptothoe kymatousa TAU-MAC 1215T [KY744812.1]
99
Leptothoe sithoniana TAU-MAC 0915T [KY744809.1]
Leptothoe spongobia TAU-MAC 1015T [KY744810.1]
71
37
Leptolyngbya sp. PCC 7375 [AB039011.1]
Adonisia turfae CCMR0081T [NR_177056.1]
73

## Slide 2
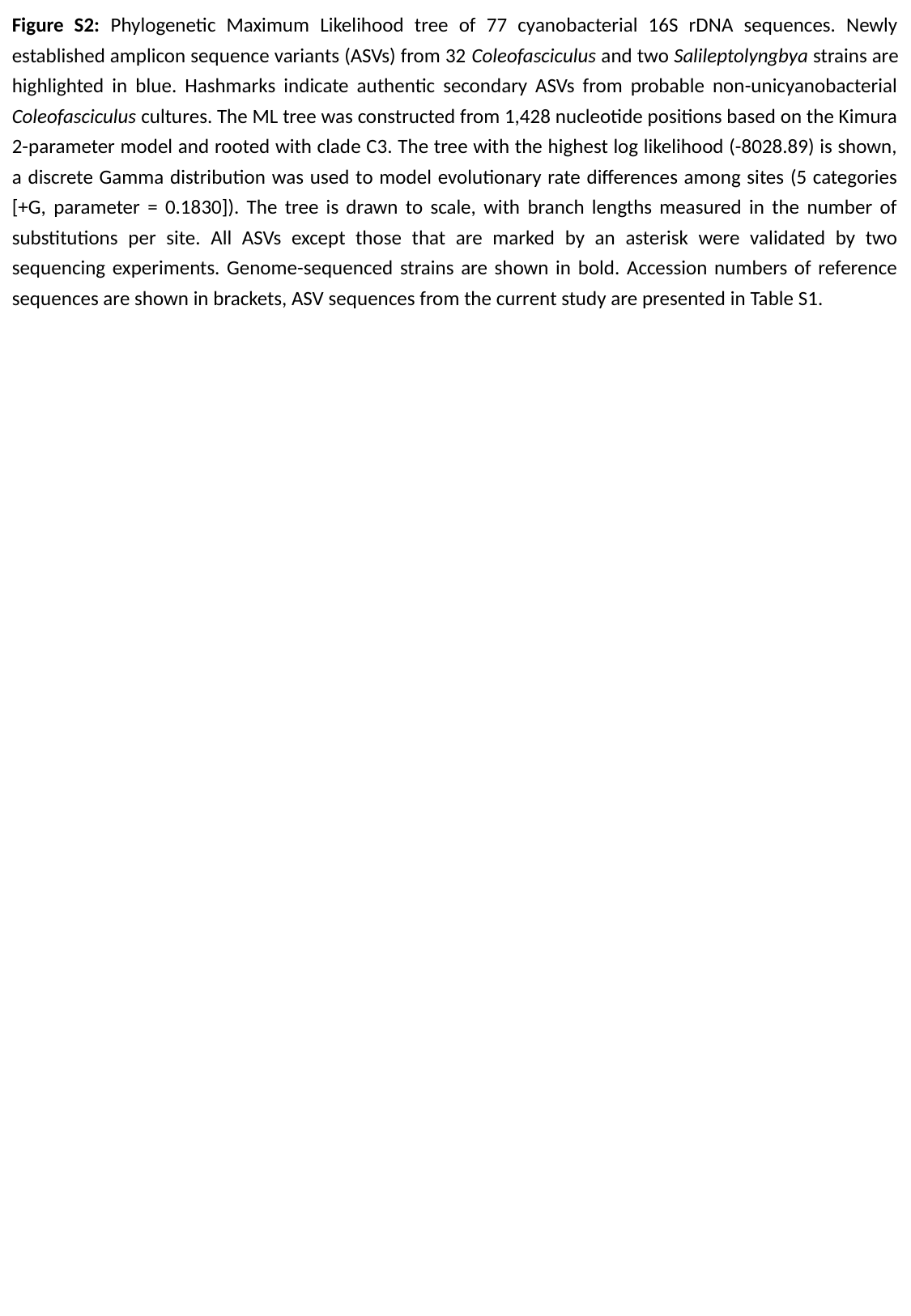

Figure S2: Phylogenetic Maximum Likelihood tree of 77 cyanobacterial 16S rDNA sequences. Newly established amplicon sequence variants (ASVs) from 32 Coleofasciculus and two Salileptolyngbya strains are highlighted in blue. Hashmarks indicate authentic secondary ASVs from probable non-unicyanobacterial Coleofasciculus cultures. The ML tree was constructed from 1,428 nucleotide positions based on the Kimura 2-parameter model and rooted with clade C3. The tree with the highest log likelihood (-8028.89) is shown, a discrete Gamma distribution was used to model evolutionary rate differences among sites (5 categories [+G, parameter = 0.1830]). The tree is drawn to scale, with branch lengths measured in the number of substitutions per site. All ASVs except those that are marked by an asterisk were validated by two sequencing experiments. Genome-sequenced strains are shown in bold. Accession numbers of reference sequences are shown in brackets, ASV sequences from the current study are presented in Table S1.
