# Supplementary material for: Superior Resolution Profiling of the Coleofasciculus Microbiome by Amplicon Sequencing of the Complete 16S rRNA Gene and ITS Region
Source: Environ Microbiol Rep. 2025 Jan 31;17(1):e70066. doi: 10.1111/1758-2229.70066 (PMC11785472; doi:10.1111/1758-2229.70066)
Supplement: Supplementary file 3 — Figure S3. Phylogenomic tree of four newly established Coleofasciculus MAGs (highlighted in blue) and 37 cyanobacterial reference genomes from clade B1, B2, B3 B4, B5 and clade A. The RaxML tree was constructed from 20,055 variable amino acid positions of 92 housekeeping genes under the GTR4Γ model and rooted with clade A. G. herdmannii, Geminocystis herdmanii; Accession numbers of reference genomes are listed in Table S8. [file EMI4-17-e70066-s005.pptx]

## Slide 1
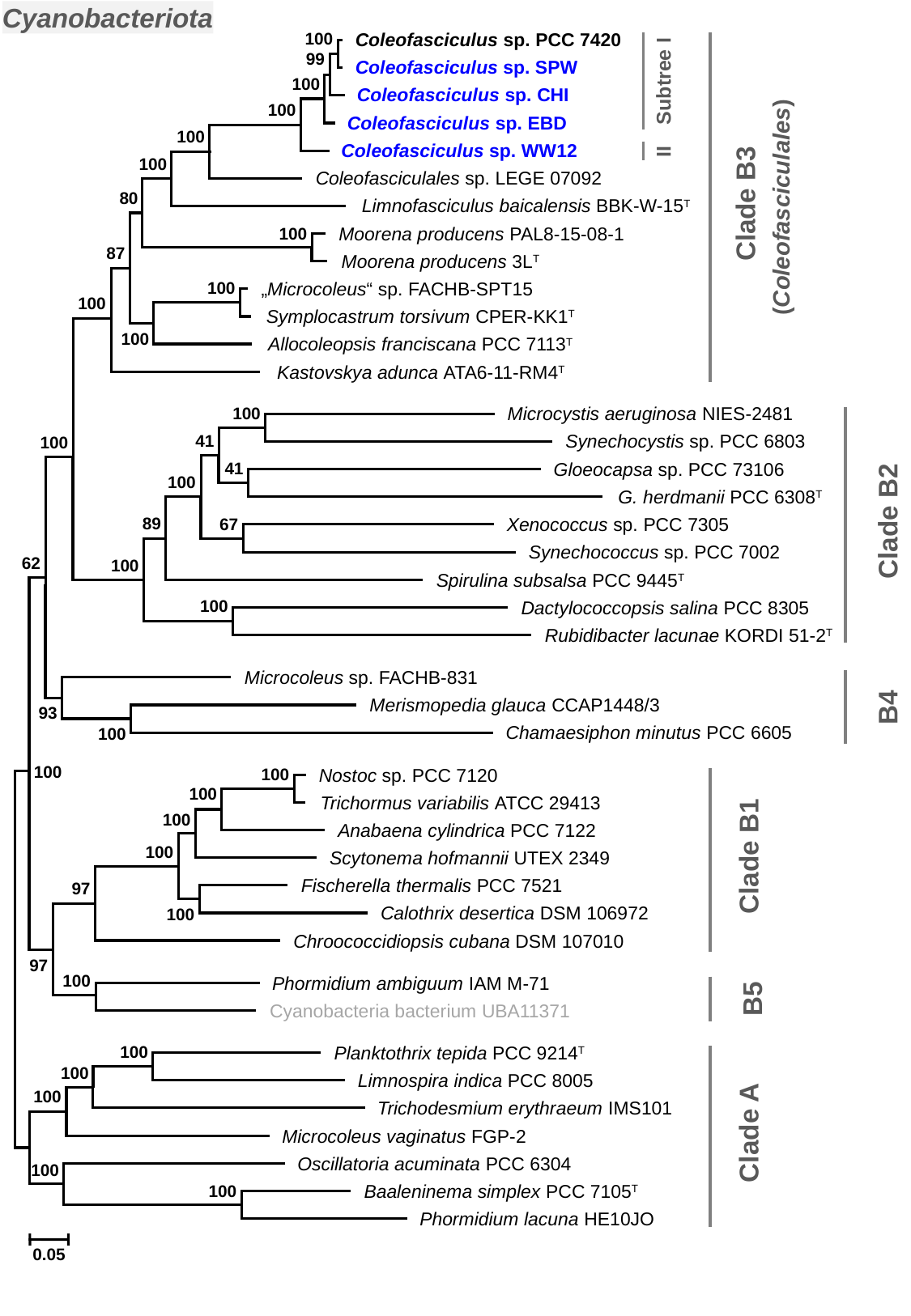

Cyanobacteriota
 Coleofasciculus sp. PCC 7420
 Coleofasciculus sp. SPW
 Coleofasciculus sp. CHI
 Coleofasciculus sp. EBD
 Coleofasciculus sp. WW12
 Coleofasciculales sp. LEGE 07092
 Limnofasciculus baicalensis BBK-W-15T
 Moorena producens PAL8-15-08-1
 Moorena producens 3LT
 „Microcoleus“ sp. FACHB-SPT15
 Symplocastrum torsivum CPER-KK1T
 Allocoleopsis franciscana PCC 7113T
 Kastovskya adunca ATA6-11-RM4T
 Microcystis aeruginosa NIES-2481
 Synechocystis sp. PCC 6803
 Gloeocapsa sp. PCC 73106
 G. herdmanii PCC 6308T
 Xenococcus sp. PCC 7305
 Synechococcus sp. PCC 7002
 Spirulina subsalsa PCC 9445T
 Dactylococcopsis salina PCC 8305
 Rubidibacter lacunae KORDI 51-2T
 Microcoleus sp. FACHB-831
 Merismopedia glauca CCAP1448/3
 Chamaesiphon minutus PCC 6605
 Nostoc sp. PCC 7120
 Trichormus variabilis ATCC 29413
 Anabaena cylindrica PCC 7122
 Scytonema hofmannii UTEX 2349
 Fischerella thermalis PCC 7521
 Calothrix desertica DSM 106972
 Chroococcidiopsis cubana DSM 107010
 Phormidium ambiguum IAM M-71
 Cyanobacteria bacterium UBA11371
 Planktothrix tepida PCC 9214T
 Limnospira indica PCC 8005
 Trichodesmium erythraeum IMS101
 Microcoleus vaginatus FGP-2
 Oscillatoria acuminata PCC 6304
 Baaleninema simplex PCC 7105T
 Phormidium lacuna HE10JO
100
99
100
100
100
100
80
100
87
100
100
100
100
41
100
41
100
89
67
62
100
100
93
100
100
100
100
100
100
97
100
97
100
100
100
100
100
100
 Clade B3
(Coleofasciculales)
 Clade B1
B5
 Clade A
Subtree I
II
 Clade B2
B4
0.05

## Slide 2
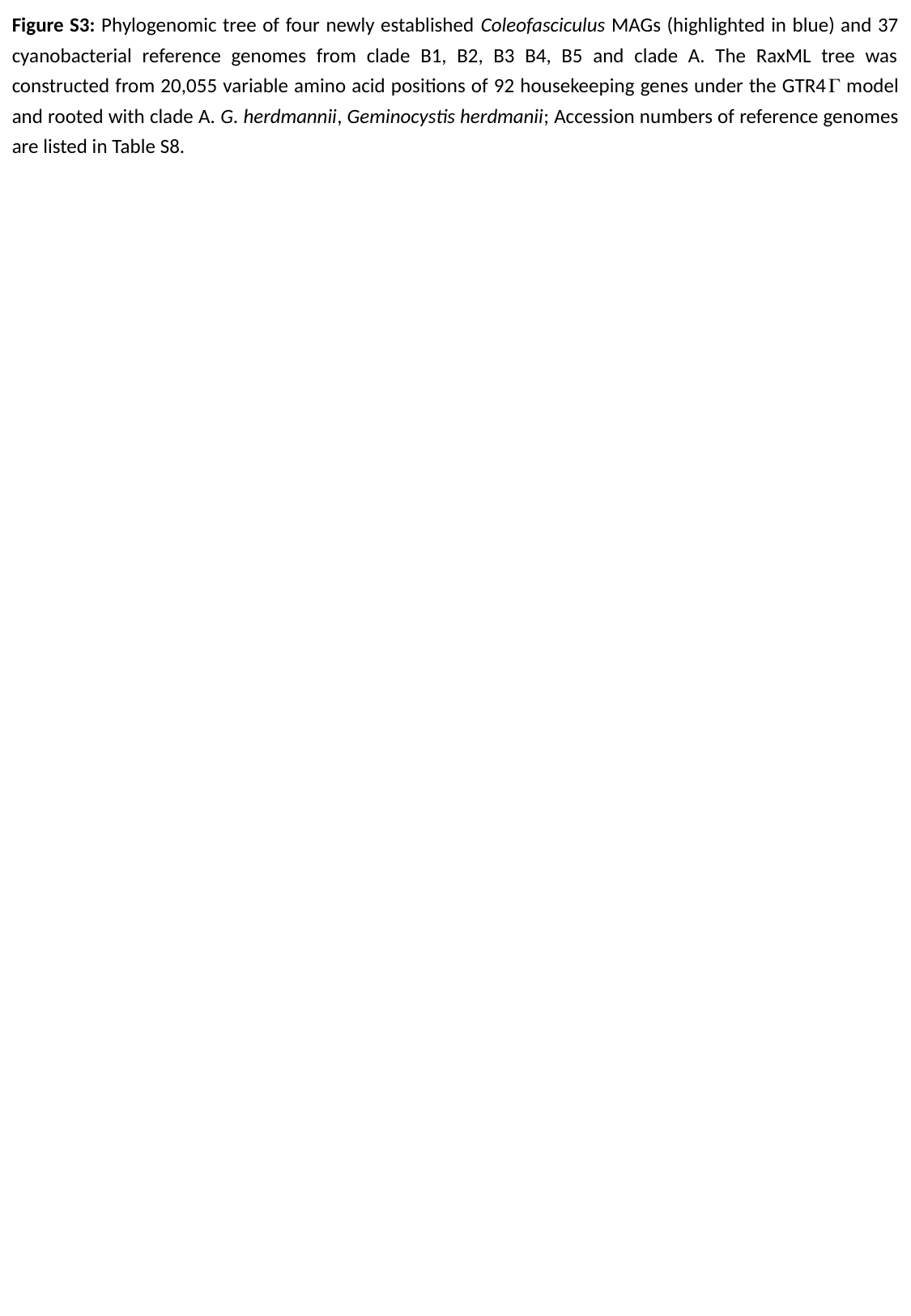

Figure S3: Phylogenomic tree of four newly established Coleofasciculus MAGs (highlighted in blue) and 37 cyanobacterial reference genomes from clade B1, B2, B3 B4, B5 and clade A. The RaxML tree was constructed from 20,055 variable amino acid positions of 92 housekeeping genes under the GTR4G model and rooted with clade A. G. herdmannii, Geminocystis herdmanii; Accession numbers of reference genomes are listed in Table S8.
