# Supplementary material for: Superior Resolution Profiling of the Coleofasciculus Microbiome by Amplicon Sequencing of the Complete 16S rRNA Gene and ITS Region
Source: Environ Microbiol Rep. 2025 Jan 31;17(1):e70066. doi: 10.1111/1758-2229.70066 (PMC11785472; doi:10.1111/1758-2229.70066)
Supplement: Supplementary file 12 — Text S1. Effect of primer barcodes and DNA sampling on 16S‐ITS amplicon sequencing. Text S2. Sequencing artefacts and contaminations. Text S3. Comparison of 16S rRNA gene phylogenies. Text S4. Genome comparison with digital DNA–DNA hybridization (dDDH). Text S5. Comparison of quantitative metagenome and amplicon data. [file EMI4-17-e70066-s009.docx]

**Supplemental Text**

**Text S1.** *Effect of primer barcodes and DNA sampling on 16S-ITS amplicon sequencing*

A comparison of the 37 authentic ASVs from *Coleofasciculus* sp. CHI showed no systematic bias from barcoding (Figure 2, Text S1). No ASV was found exclusively in the BC018 datasets, and the use of BC002 resulted in only one low-abundance sequence (ASV-113) that was specific for this barcode. By contrast, sampling the DNA four years apart resulted in the occurrence of several unique ASVs; three 16S-ITS sequences were specific to the sample from 2019 (ASV-287, - 412, -005) and five ones to those from 2023 (ASV-443, -407, -232, -650, -130). However, long-read sequencing was not performed to saturation, so it is unlikely that this pattern reflects true losses of associated bacteria or secondary contamination, but can most probably be explained by changes in the abundance of the accompanying bacteria. Amplicon sequencing revealed a diverse composition of the *Coleofasciculus* sp. CHI cyanosphere with dozens of associated heterotrophic bacteria. Their abundance is probably influenced by abiotic factors and the age of the cyanobacterial culture, leading to an individual composition of the isolated metagenomic DNA and thus to changes in PCR amplification. General abundance comparisons showed that these differences are typical for most of the strains examined (Figure 3, Figure S1). Very low sequence counts of e.g. the *Thalassospiraceae* bacterium ASV-005 (7x) and the *Reichenbachiellaceae* bacterium ASV-412 (15x) in 2019 therefore probably fell below the detection limit in the CHI sample from 2023.

**Text S2.** *Sequencing artifacts and contaminations*

Singleton ASVs detected only in one experiment should be treated with special caution as they could represent PCR artifacts or sporadic contaminations. (i) A typical PCR error was detected in the low abundant singleton ASV-981 from *Coleofasciculus* sp. CHI (16 sequence counts, BC002 [2023]; Table S1). Its sequence with a size of 2,029 bp contains a single point mutation (T:A) compared to the third most common ASV-439 from *Ekhidna* sp. (4,150 sequence counts), which was found in all four sequencing runs. The two orders of magnitude lower abundance of this singleton compared to the authentic ASV likely reflects a PCR error that occurred regardless of the use of ultrahigh-fidelity Platinum^TM^ SuperFi^TM^ DNA polymerase. The detection of these very rare errors corresponds to the depth of high-throughput sequencing, but their frequency could even be reduced by decreasing the number of PCR cycles (Sze and Schloss, 2019). (ii) A second category of typical PCR artifacts are chimeric amplicons. The singleton ASV-1094 from strain EBD with only four sequence counts is a chimera of two authentic 16S-ITS sequences, the cyanobacterial ASV-604 from *Coleofasciculus* sp. and the alphaproteobacterial ASV-117 from *Stappia indica* (Table S1). This chimeric sequence, 2,095 nt in length, passed our stringent filtering pipeline due to double recombination; the 5' and 3' ends are identical to the *Coleofasciculus* ASV and the region from nucleotide 227 to 861 corresponds to *Stappia*. Accordingly, promising singletons should be individually checked by parallel BLASTN searches of different regions of the 16S rRNA gene to rule out the presence of chimera. (iii) Finally, it should be kept in mind that PCR-amplification combined with high-throughput sequencing allows the detection of even minimal contaminations in reagents (Salter *et al.*, 2014; Eisenhofer *et al.*, 2019). In the current study cyanobacterial harvesting for DNA extraction and all pipetting steps for PCR amplification were performed with gloves under the clean bench. The presence of 34 authentic ASVs in the *Coleofasciculus* sp. CHI sample BC002 (2023) reflects the quality of the large dataset (Figure 2), but the detection of 21 betaproteobacterial ASVs among the singletons in this sequencing run was indicative of DNA contamination in the reagents due to the generally sparse occurrence of *Betaproteobacteria* in marine samples (Table S1). However, all *Variovorax* and *Acidovorax* (*Burkholderiales*, *Betaproteobacteria*) ASVs from the current study detected in high-throughput sequencing data of seven of the 34 strains examined (CHI, STO, NDN, GNP5, WW1, WW9, EDA) were singletons. Accordingly, validation of amplicon sequencing by two independent runs allowed efficient filtering of sporadic contaminations, and it was the basis for the identification of authentic ASVs without any PCR errors.

**Text S3.** *Comparison of 16S rRNA gene phylogenies*

The 16S rRNA gene tree in the current study (Figure 4) is largely congruent with the previous phylogeny of Siegesmund et al. (Siegesmund *et al.*, 2008), but we detected three inconsistencies: (i) The previous study proposed a moderately supported association of BRE with sequences of subtree II (Siegesmund *et al.*, 2008), whereas our investigation clearly documents its localization in subtree I with a common branching of the two unique BRE sequences (ASV-602, ASV-599) together with ASVs from EBD, WW3, WW5 and WW6 (Figure 4B). We observed several sequence mismatches (Table S3) that may reflect the non-identity of the deposited 16S rRNA gene or previous sequencing errors. The authenticity of the newly established ASVs of strain BRE, which was deposited in our culture collection as *Coleofasciculus* sp. DSM 104253, was confirmed by identical sequencing results of DNA samples extracted in 2019 and 2023 (Table S1). (ii) The basal positioning of GNP5 and SOL in subtree I (Figure 4B), which was not observed in the former analysis (Siegesmund *et al.*, 2008), likely reflects a long branch attraction artifact of these 16S sequences due to an accelerated evolution rate (Philippe *et al.*, 2005). Phylogenomic analyses would be required to confirm the common branching of strains GNP5 and SOL and to determine their exact position in the *Coleofasciculus* subtree I. (iii) The previously reported 100% 16S-rRNA identity of WW1, WW4, WW8, WW10 and WW12 with *Sodalinema stali* SAG 31.92 (*Oscillatoriales*; EF654088.1 (Siegesmund *et al.*, 2008)) was inconsistent with values of less than 92% observed for authentic ASVs established in the current study. Three isolates, namely WW1, WW10 and WW12, could be classified as *Coleofasciculus* strains of subtree II (*Coleofasciculales*, Clade B3), while the two remaining isolates with identical ASVs, namely WW4 and WW8, were relatives of *Salileptolyngbya diazotrophica* SCSIO 43686 (*Nodosilinales*, Clade C3; Figure S2).

**Text S4.** *Genome comparison with digital DNA-DNA hybridization (dDDH)*

Comparison of the four newly established high-quality *Coleofasciculus* MAGs and the reference genome of strain PCC 7420 using digital DNA-DNA hybridization (dDDH) with the Genome-to-Genome Distance Calculator (GGDC) (Meier-Kolthoff *et al.*, 2013) yielded values below 40% for strain WW12 of subtree II (Table S5), confirming the 16S rDNA-based conclusion that WW12 represents a second *Coleofasciculus* species (threshold: 70% (Meier-Kolthoff *et al.*, 2013)). The low dDDH values correlated with an average nucleotide identity (ANI) of only 88%. The closely related Woods Hole strains PCC 7420 and SPW belong to the same species (dDDH: 83.7%, ANI: 97.9%; (Stackebrandt and Ebers, 2006; Jain *et al.*, 2018)), but dDDH values of less than 55% and ANI values below 93% suggest that CHI and EBD are representing two other closely related species of subtree I (Figure 5C). According to the criteria for species delineation of bacteria and the classification of cyanobacteria according to the International Code of Nomenclature of Prokaryotes (ICNP; the “Prokaryotic Code” (Oren and Ventura, 2017)), the five genome-sequenced strains are likely members of four species, and the diversity of the 16S rDNA phylogeny indicates the presence of additional cryptic species in the genus *Coleofasciculus*.

**Text S5.** *Comparison of quantitative metagenome and amplicon data*

Mapping of raw reads from metagenome sequencing on the identified MAGs allowed a determination of the individual genome coverages, which should reflect the relative abundance of bacteria in the cyanosphere (Figure 5A, Table S2). However, a comparison of ASV and metagenome classification on phylum level revealed major quantitative differences in the composition of the amplicon data beyond the absence of *Planctomycetota* (Figure 5B). A striking disparity is the proportion of cyanobacterial ASVs, which are overrepresented by a factor of three in EBD and SPW but almost absent in WW12. Other examples are the *Balneolota* in CHI, the *Alphaproteobacteria* in EBD, and the *Actinomycetota* in WW12. The observed differences could be related to different numbers of the ribosomal operon in the bacterial genomes. However, the most likely explanation for the non-representative amplicon data is biased PCR-amplification due to individual primer binding correlating with the genomic G+C-content (37-72%; Table S2) and differences in amplicon length of up to one kilobase pair (CHI: 1,827 bp – 2,804 bp; Table S7). The comparison of different 16S-ITS amplicon sequencing experiments based on the same DNA sample showed large quantitative differences in long-read amplicon sequencing (Figure S1), which might be related to the different primer barcodes used. In addition, previous analyses of artificial mock communities have shown that the 16S-ITS amplicon data do not reliably reflect the proportion of bacteria (unpublished data).

**References**

Eisenhofer, R., Minich, J.J., Marotz, C., Cooper, A., Knight, R., and Weyrich, L.S. (2019) Contamination in low microbial biomass microbiome studies: Issues and recommendations. *Trends Microbiol* **27**: 105–117.

Jain, C., Rodriguez-R, L.M., Phillippy, A.M., Konstantinidis, K.T., and Aluru, S. (2018) High throughput ANI analysis of 90K prokaryotic genomes reveals clear species boundaries. *Nat Commun* **9**: 5114.

Meier-Kolthoff, J.P., Auch, A.F., Klenk, H.-P., and Göker, M. (2013) Genome sequence-based species delimitation with confidence intervals and improved distance functions. *BMC Bioinformatics* **14**: 60.

Oren, A. and Ventura, S. (2017) The current status of cyanobacterial nomenclature under the “prokaryotic” and the “botanical” code. *Antonie van Leeuwenhoek, Int J Gen Mol Microbiol* **110**: 1257–1269.

Philippe, H., Zhou, Y., Brinkmann, H., Rodrigue, N., and Delsuc, F. (2005) Heterotachy and long-branch attraction in phylogenetics. *BMC Evol Biol* **5**: 1–8.

Salter, S.J., Cox, M.J., Turek, E.M., Calus, S.T., Cookson, W.O., Moffatt, M.F., et al. (2014) Reagent and laboratory contamination can critically impact sequence-based microbiome analyses. *BMC Biol* **12**: 87.

Siegesmund, M.A., Johansen, J.R., Karsten, U., and Friedl, T. (2008) Coleofasciculus gen. nov. (Cyanobacteria): Morphological and molecular criteria for revision of the genus Microcoleus Gomont. *J Phycol* **44**: 1572–1585.

Stackebrandt, E. and Ebers, J. (2006) Taxonomic parameters revisited: tarnished gold standards. *Microbiol Today* **33**: 152–154.

Sze, M.A. and Schloss, P.D. (2019) The impact of DNA polymerase and number of rounds of amplification in PCR on 16S rRNA gene sequence data. *mSphere* **4**: e00163-19.
